# Hotspot Mutations in KIT Receptor Differentially Modulate Its Allosterically Coupled Conformational Dynamics: Impact on Activation and Drug Sensitivity

**DOI:** 10.1371/journal.pcbi.1003749

**Published:** 2014-07-31

**Authors:** Isaure Chauvot de Beauchêne, Ariane Allain, Nicolas Panel, Elodie Laine, Alain Trouvé, Patrice Dubreuil, Luba Tchertanov

**Affiliations:** 1 Bioinformatics, Molecular Dynamics & Modeling (BiMoDyM), Laboratoire de Biologie et Pharmacologie Appliqués (LBPA-CNRS), Ecole Normale Supérieure de Cachan, Cachan, France; 2 Centre de Mathématiques et de Leurs Applications (CMLA-CNRS), Ecole Normale Supérieure de Cachan, Cachan, France; 3 Inserm, U1068, Signaling, Hematopoiesis and Mechanism of Oncogenesis (CRCM); Institut Paoli-Calmettes; Aix-Marseille University; CNRS, UMR7258, Marseille, France; Institut Pasteur, France

## Abstract

Receptor tyrosine kinase KIT controls many signal transduction pathways and represents a typical allosterically regulated protein. The mutation-induced deregulation of KIT activity impairs cellular physiological functions and causes serious human diseases. The impact of hotspots mutations (D816H/Y/N/V and V560G/D) localized in crucial regulatory segments, the juxtamembrane region (JMR) and the activation (A-) loop, on KIT internal dynamics was systematically studied by molecular dynamics simulations. The mutational outcomes predicted *in silico* were correlated with *in vitro* and *in vivo* activation rates and drug sensitivities of KIT mutants. The allosteric regulation of KIT in the native and mutated forms is described in terms of communication between the two remote segments, JMR and A-loop. A strong correlation between the communication profile and the structural and dynamical features of KIT in the native and mutated forms was established. Our results provide new insight on the determinants of receptor KIT constitutive activation by mutations and resistance of KIT mutants to inhibitors. Depiction of an intra-molecular component of the communication network constitutes a first step towards an integrated description of vast communication pathways established by KIT in physiopathological contexts.

## Author Summary

Receptor tyrosine kinase KIT plays a crucial role in the regulation of cell signaling. This allosterically controlled activity may be affected by gain-of-function mutations that promote the development of several cancers. Identification of the molecular basis of KIT constitutive activation and allosteric regulation has inspired computational study of KIT hotspot mutations. In the present contribution, we investigated the mutation-induced effects on KIT conformational dynamics and intra-protein communication conditionally on the mutation location and the nature of the substituting amino acid. Our data elucidate that all studied mutations stabilize an inactive non-autoinhibited state of KIT over the inactive auto-inhibited state prevalent for the native protein. This shift in the protein conformational landscape promotes KIT constitutive activation. Our *in silico* analysis established correlations between the structural and dynamical effects induced by oncogenic mutations and the mutants auto-activation rates and drug sensitivities measured in vitro and in vivo. Particularly, the A-loop mutations stabilize the drug-resistant forms, while the JMR mutations may facilitate inhibitors binding to the active site. Cross-correlations established between local and long-range structural and dynamical effects demonstrate the allosteric character of the gain-of-function mutations mode of action.

## Introduction

**Figure 1:**
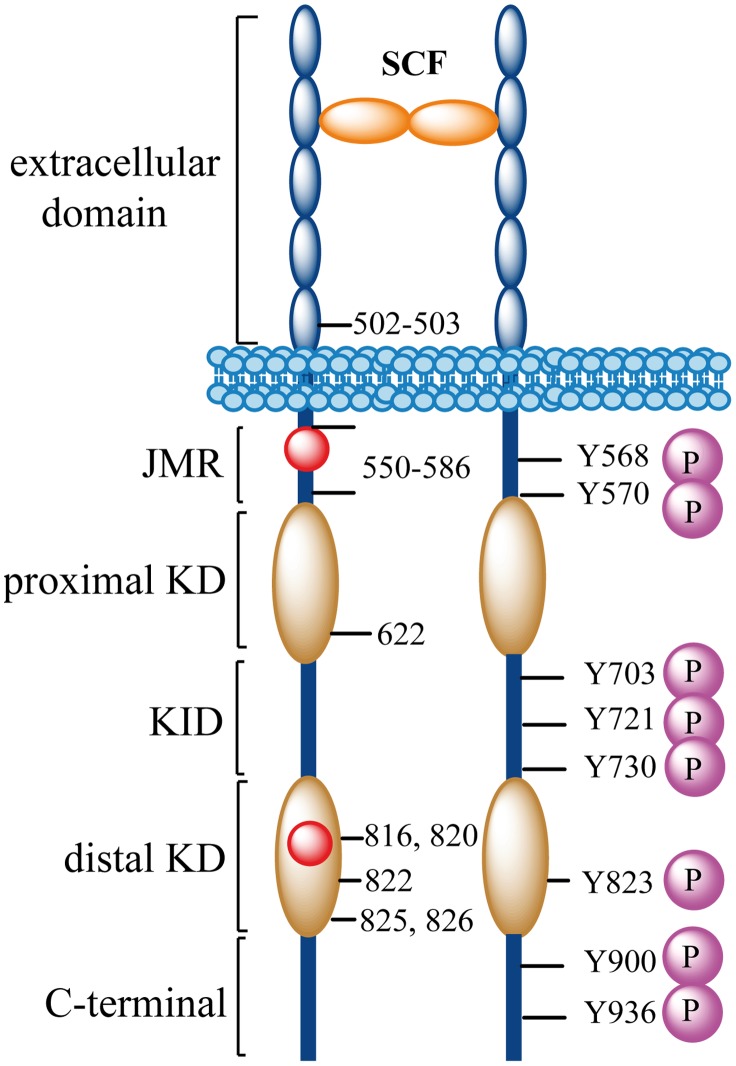
Structural organization of KIT. Stem Cell Factor (SCF) binding on the extracellular domain of KIT induces dimerization. Upon activation, KIT is autophosphorylated at tyrosine residues (magenta balls) that act as binding sites for downstream signaling kinases/mediators. The location of KIT gain-of-function mutations is indicated by residue numbers. Residues V560 and D816 (red balls), positioned in the JMR and in the kinase domain of the cytoplasmic region, are associated with highly malignant cancers. JMR, juxta-membrane region; KD, kinase domain; KID, kinase insert domain.

The transmembrane receptor tyrosine kinase (RTK) KIT, also known as Stem Cell Factor (SCF) receptor, plays a key role in cell survival, differentiation, maturation and function [1], [2]. KIT belongs to the subfamily of RTK type III characterized by the presence of five Ig-like subunits in their extracellular domain. This extracellular portion contains the ectodomain with a ligand (SCF for KIT) binding site and is linked to a cytoplasmic region by a single transmembrane helix (**Figure 1**). The cytoplasmic region consists of an autoinhibitory juxta-membrane region (JMR) and a kinase domain (KD) arranged in a proximal (N-) and a distal (C-) lobes linked by a hinge region. The distal lobe of RTKs type III includes a large Kinase Insert Domain (KID) of ∼60–100 residues.

**Figure 2:**
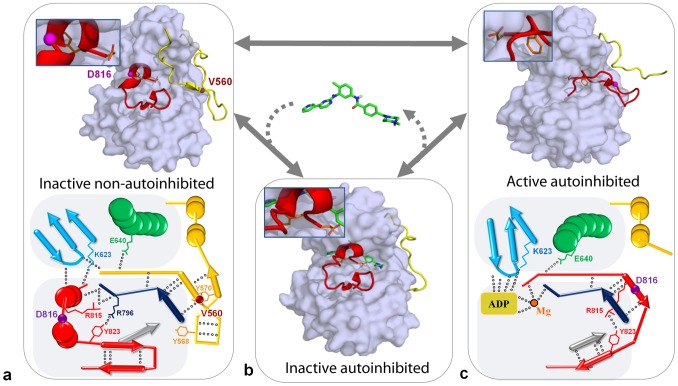
The inactive-to-active state switch of KIT tyrosine kinase domain. (**a**) The inactive auto-inhibited state with the A-loop and the JMR adjacent to the active site (1T45 [34] (**c**) and the active state with a solvent-exposed JMR and an extended A-loop conformation (1PKG [108]) with schematic representation of the secondary structures observed in the inactive and the active states. (**b**) The intermediate state, inactive not auto-inhibited, observed in KIT complex with imatinib (1T46 [34]). Protein surface of KIT (gray) with the A-loop (red) and the JMR (yellow) shown as cartoons. Imatinib is shown as sticks. The principal H-bonds stabilizing the β-sheets are shows by green doted lines. Positions of residues V560 and D816, if resolved, are indicated as balls. The catalytic residues, DFG, indicated as sticks are zoomed in the inserts.

In absence of SCF, KIT monomer is in equilibrium between different states: the inactive auto-inhibited state that is highly dominant, the intermediate states, and the active state (**Figure 2**) [3]. The binding of SCF to the ectodomain of the receptor induces interactions between two KIT monomers, leading to receptor dimerization which initiates the transphosphorylation of specific tyrosine residues [1], [2], [4], [5]. Once phosphorylated, these tyrosine residues serve as binding sites for the recruitment of downstream signaling proteins which regulate specific signaling cascades, crucial for the control of cellular activities such as proliferation or apoptosis [6]–[8]. In human KIT, the KID (77 residues) and the JMR (35 residues) contain three and two phosphorylated tyrosine residues, respectively [5].

The active site of the kinase domain is localized between the two lobes. Two crucial regulatory segments, the activation loop (A-loop, residues 810–835) and the JMR (residues 547–581), undergo extensive conformational rearrangements during the activation/deactivation processes. In the inactive auto-inhibited state of the receptor, the A-loop is adjacent to the active site, and the highly conserved catalytic D810-F811-G812 (DFG) motif at its N-extremity adopts an “out” conformation: the phenylalanine is flipped into the ATP-binding site, thus impairing ATP and Mg^2+^ cofactor binding [9], [10]. This conformation is stabilized by the JMR that inserts itself directly into the kinase active site and prevents the A-loop to adopt an active conformation. Upon activation, the JMR moves from its auto-inhibitory position to a completely solvent-exposed emplacement. This intermediate conformation presenting an inactive, but not auto-inhibited state of the kinase domain, is followed by a conformational swap of the A-loop from an inactive packed arrangement to an active extended conformation (**Figure 2**). Such structural transformations together with a switch of the DFG motif to an “in” conformation favors the KIT kinase domain transition to the active state which allows ATP entrance and binding in the catalytic site.

Several gain-of-function point mutations induce KIT constitutive (SCF-independent) activation. Such mutations are responsible for abnormal cell expression resulting in numerous proliferating diseases such as mastocytosis [11], gastro-intestinal stromal tumor (GIST), and leukemia [1], [5], [6], [12]. These mutations were identified in the membrane-proximal extracellular domain, in the JMR and in the kinase domain (**Figure 1**). The JMR mutations, particularly at positions V559–V560, have been associated with human GISTs [12]–[14]. KIT constitutive activation induced by mutations V560D and V560G was evidenced *in vitro* and *in vivo* [15]–[17]. These JMR mutants have been considered as deregulatory as they disrupt the autoinhibitory function of the JMR. Mutations in the kinase domain, frequently observed in the A-loop in cases of mastocytosis, AML [18]–[20] and human germ cell tumors [21], are believed to strongly influence the A-loop conformation, contributing in the displacement of KIT equilibrium towards the active conformation. The most frequently observed mutation consists in an amino acid substitution at position 816 in KIT A-loop from an aspartic acid (D) to a valine (V) [22], [23], a histidine (H) [24], an asparagine (N) [25] or a tyrosine (Y) [22], [26], [27]. These mutants were shown to increase kinase activity and transforming activity *in vitro* and *in vivo* [14], [18], [28], [29]. The experimentally measured activation rate of KIT mutants depends on the localization of the mutation and on the type of substituting amino acid [30]. In particular, the D816V and D816H mutants auto-activate substantially faster than native KIT in kinase activity assays (586- and 184-fold, respectively). The auto-activation rate was even higher (>600 fold) for KIT mutant V560D.

Auto-activated mutants of kinases define a clinically validated class of targets for cancer drug therapy [31]. Therefore, enormous efforts are devoted to the development of small molecules (inhibitors) targeting oncogenic kinases activity. However, extensive studies have revealed that many kinase inhibitors are less selective than initially expected [32], [33]. The kinase inhibitor imatinib (Gleevec^(R)^ or Glivec^(R)^) is highly specific for a restricted number of kinases (recombinant receptor Bcr-Abl, and receptors of type III KIT and PDGFR). Structural data revealed that imatinib binds to the active site of the receptor and maintains the native KIT in an inactive non-autoinhibited state (PDB ids: 1T46, 1.60 Å resolution [34]). Such mode of binding denotes imatinib as an ATP competitor. Imatinib also inhibits KIT mutants associated with GIST, in particular those carrying somatic mutations in the JMR. KIT mutants V560G/D (in JMR) are remarkably sensitive, even more than the native KIT, to imatinib [15], [35], [36]. In contrast, imatinib is poorly efficient as an inhibitor of KIT mutants D816V/H/Y/N found in mastocytosis [36].

Acquired resistance to systemic therapy is a serious obstacle to the treatment of metastatic cancers. A representative example of acquired resistance is given by KIT secondary mutations identified in naïve GIST patients treated with imatinib [37]. These mutations are positioned either in the ATP-binding pocket or in the kinase activation loop [38]. The second-line treatment, after imatinib failure, is provided by less specific kinase inhibitors, such as sunitinib that shows potency against imatinib-resistant KIT mutated in the ATP-binding pocket (V654A and T670I) [39]. In contrast, some imatinib-resistant mutants, including D816V/H, are also resistant to those second-line inhibitors [35].

The reported high-resolution crystal structures of native RTKs provide a molecular basis for understanding their catalytic activity and their physiological activation mechanisms. Beyond the fundamental knowledge about these receptors' enzymatic function, structural data also furnish a vision on RTKs inhibition mechanisms and constitute a crucial basis for the rational design of inhibitors capable to interfere with RTKs catalytic activity. By contrast, the mechanisms of RTKs constitutive activation caused by gain-of-function mutations are not yet fully described. Particularly, the structural characterization of KIT mutants is limited. Although some structural details related to KIT D816H mutant were reported [30], there is no direct dynamical evidence to support the mechanisms of a constitutive activation in KIT mutants.

To contribute in the elucidation of the impact of mutations on KIT mechanisms of activation and resistance to anticancer drugs, we performed a systematic analysis of the structural and dynamical effects induced by point mutations localized in the two major regulatory fragments of KIT – the A-loop and the JMR – on KIT cytoplasmic region. In our molecular dynamics (MD) simulations a set of clinically observed mutations (D816H/Y/N/V, V560G/D) was considered and the impact of each mutation was explored. Such study of KIT mutants at the atomic level represents a triple interest. First, it should contribute to a better understanding of kinases constitutive activation mechanisms. Second, it helps to distinguish between the contributions of primary (activation) and secondary (resistance) mutations on KIT activity and drug sensitivity. Third, the produced data are of crucial importance for the identification of novel relevant therapeutic targets and for providing guidance to the design of novel drugs highly specific to KIT.

We have recently reported the first step of this study, a comparison of structural, dynamics and thermodynamics features of the native KIT and its D816V mutant [40]. We have evidenced that the D816V mutation induces two main structural effects on KIT kinase domain: a local effect expressed by a partial unfolding in the A-loop at proximity of the mutation site and a long-range effect manifested by an important structural reorganization of the JMR, yet distant by more than 15 Å from the point mutation. We demonstrated that the structural reorganization of the JMR in KIT mutant is a result of the perturbation of a communication pathway between the A-loop and the JMR observed in the native protein [41]. Here we report MD simulations of KIT mutants harbouring either D816H/Y/N or V560G/D mutation. The results of the simulations were carefully analyzed and compared with those found for the native KIT and its D816V mutant, and with reported experimental data.

## Materials and Methods

### Molecular dynamics simulations

#### Initial coordinate files

The crystallographic coordinates for the wild-type (WT) KIT (PDB id: 1T45, 1.90 Å resolution, no co-crystallized ligand) [34] was retrieved from Protein Data Bank (PDB) [42] and used as a template for modeling KIT V560G/D and D816H/Y/N mutants. The crystallographic structure contains residues from 547 to 935 with the exception of a large part of the KID (residues 693–753) that was replaced by a short linker. As reported in all crystallographic studies of type III RTKs, such a deletion was applied systematically to successfully achieve the protein crystallization, strongly affected by a high mobility of the KID domain [43]. The shortened engineered KID, further referred to as “*pseudo*-KID”, will be neglected in the analyses. All crystallographic water molecules were removed. Five initial models were generated: (**I**) KIT^V560G^, (**II**) KIT^V560D^, (**III**) KIT^D816Y^ and (**IV**) KIT^D816N^, were built with MODELLER 9v7 [44], [45], and (**V**) KIT^D816H^, with Maestro 9.1 from Schrödinger Suite [46], using a protocol identical to that previously employed for the KIT^WT^ and KIT^D816V^ modeling [40]. All models were prepared using the LEAP module of AMBER 10 [47] with the *parm99* parameter set: (i) hydrogen (H) atoms were added, (ii) covalent bond orders were assigned, (iii) Na^+^ counter-ions were added to neutralize the system charge, (iv) the protein was hydrated in a periodic box of explicit TIP3P water molecules [48]. A hydration shell of 9 Å was employed for D816H/Y/N mutants. This distance was increased up to 12 Å for V560G/D mutants, as we anticipated for large motions of the JMR in these mutants, according to the experimental data reporting an increased solvent accessibility and fluctuations of the JMR in KIT mutated in this region [49]. The preparation details of the MD simulations are given in **Table S1**.

#### Protonation state of H816

The histidine H816 in KIT^D816H^ was protonated on the N^δ1^ atom as this protonation state corresponded to an optimized H-bond network according to Maestro evaluation. A close inspection of H816 environment and solvent accessibility [50] further supported this choice. The KIT^D816H^ model was then minimized using MacroModel [46].

#### Set up of the systems

Set up of the systems was performed with SANDER module of AMBER 10 [47]. First, each system was minimized successively by steepest descent and conjugate gradient algorithms [51] as follows: (i) 10 000 steps of minimization of the water molecules keeping protein atoms fixed, (ii) 10 000 steps of minimization keeping only protein backbone fixed, to allow protein side chains to relax, and (iii) 10 000 steps of minimization without any constraint on the system. After relaxation, each system was gradually heated from 10 to 310 K at constant volume using the Berendsen thermostat [52] while restraining the solute Cα atoms by 10 kcal/mol/Å^2^. Thereafter, the system was equilibrated for 100 ps at constant volume (NVT) and for further 100 ps at constant pressure (NPT) maintained by a Langevin piston [53]. Finally, the restraints were removed and each system was equilibrated for a final 100-ps run. Backbone deviations obtained after equilibration are reported in **Table S1**.

#### Production of the trajectories

For each equilibrated system (models **I**–**V**) two independent MD simulations were run with different initial velocities using the PMEMD module of AMBER 10. The temperature was kept at 310 K (Berendsen thermostat), and pressure at 1 bar (Langevin piston coupling algorithm). The SHAKE algorithm was used to freeze covalent bonds involving hydrogen atoms, allowing for an integration time step of 2.0 fs. Long-range electrostatic interactions were treated by the Particle Mesh Ewald method [54]. Each simulation run was continued until a total time of 50 ns to 70 ns, depending on the estimated convergence of the first 50 ns of simulation. Coordinates files were recorded every 1 ps.

#### Analysis of the trajectories

Unless otherwise stated, all recorded MD trajectories were analyzed with the PTRAJ module of AMBER 10. From the Root Mean Square Deviation (RMSD) profiles, 2 ns (for KIT^D816Y/N^) or 5 ns (for KIT^D816H^ and KIT^V560G/D^) were found sufficient to achieve relaxation. To compare simulations of equal “productive” duration in all KIT^D816V/H/Y/N^ mutants, we extended KIT^D816H^ simulations until 53 ns. The last 48 (KIT^D816V/H/Y/N^) or 65 ns (KIT^V560G/D^) were consequently retained for further analysis.

#### Convergence analysis

A convergence analysis was performed on the productive simulation time of each MD trajectory (2×48-ns or 2×65-ns) using an ensemble-based approach [55]. The algorithm extracts representative MD conformations from a trajectory by clustering the recorded snapshots according to their Cα-atoms RMSDs. The procedure for each trajectory can be described as follows: (i) a *reference* structure is randomly picked up in the MD conformational ensemble and all conformations distant by less than an arbitrary cutoff *r* are popped out of the ensemble; this step is repeated until no conformation remains in the ensemble, providing a set of *reference* structures distant from at least *r*; (ii) the MD conformations are grouped into *n reference* clusters based on their RMSDs from each *reference* structure; (iii) in each *reference* cluster the number of conformations coming from the 1^st^ - or the 2^nd^ half of the simulation are counted and compared. The closer these two numbers in each cluster, the more converged the simulation, meaning that each *reference* conformation is visited regularly during the simulation. The RMSD cutoff *r* was empirically chosen so as to best capture the conformational diversity of the MD conformational ensemble leading to 3–5 representative MD conformations. To avoid or to diminish the bias resulting from the random choices of the *references*, the process was repeated 5 times for each analyzed trajectory. To quantitatively evaluate and compare the convergence quality of the produced MD simulations, we defined a convergence criterion *c* as the average ratio of the number of *lone reference* structures (visited only in one half of the trajectory) over the total number of *reference* structures:
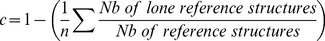
(1)where 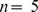 is the number of runs performed. The result is comprised between 0 and 1; a value of 1 corresponds to an optimal convergence, when each *reference* structure is represented by conformations from both halves of the trajectory.

This method selected 5 different sets of representative MD conformations for each trajectory and the set being the most equally represented in the 4 halves of simulation (2 halves per replica) was retained for further analysis.

#### Clustering of the A-loop conformations from the MD simulations

The conformational variability of the A-loop in KIT^WT^ and KIT^D816H/Y/N/V^ mutants was analyzed along the MD simulations by applying a clustering method similar to the one described above. The MD conformations of all 5 models of KIT were superimposed and clustered according to RMSDs computed on the backbone atoms of the A-loop. To improve the robustness of the statistical analysis, 5 independent clustering runs were performed, producing 5 different sets of representative MD conformations of the A-loop. In each cluster, the number of conformations issued from each MD simulation was counted. To obtain a reasonable number (4 - 8) of representative MD conformations, the RMSD cutoff *d* was empirically set to 2.5 Å. A similar analysis was performed to estimate the conformational variability of the JM-Switch in KIT^WT^ and KIT^D816H/Y/N/V^ and KIT^V560G/D^ mutants, with *d* = 4 Å.

#### Secondary and tertiary structure

Secondary structures were assigned every 1 ps using DSSP method [56]. Time occupancies of H-bonds stabilizing the JMR and the A-loop were recorded every 100 ps. H-bonds (D–H•••A) were defined with a DHA angle cutoff of 120° and a D•••A distance cutoff of 3.5 Å. Several characteristic distances and angles were monitored every 10 ps: (i) the distance D_JMS_ between the center of mass (CoM) of the JM-Switch (residues 560–570) and the CoM of the two closest residues in the C-lobe (847 and 912); (ii) the angle A_JMS_ formed by the CoM of the JM-Switch, the CoM of the protein KD (residues 582–935, except for the *pseudo*-KID, 694–762) and the CoM of the C-lobe (residues 763–935); (iii) the distance D_A_ between the CoM of the A-loop (residues 210–239) and the rest of the KD, excluding the *pseudo*-KID.

#### Secondary structures prediction

The secondary structures of the 20 amino acids polypeptide (residues 555–575) constituting the JMR of KIT were predicted by using different methods: GOR4 [57], SOPMA [58], SIMPA96 [59], PREDATOR [60], PHD [61] and Jnet [62]. We combined them to increase the statistical meaning of the predictions, and a score was assigned to every secondary structure motif (β-strand, α-helix, coil and turns) for each amino acid.

#### Principal component analysis

A Principal Component Analysis (PCA) was applied to identify the eigenvectors (3N degrees of freedom) describing the main collective motions of the protein. The calculations were performed on the backbone atoms positions along the simulation production time 96 ns in total for KIT^D816H/Y/N/N^ mutants, and 130 ns for KIT^V560G/D^ mutants. The degree of collectivity *k* of a domain D in a mode *m* was calculated either directly (2a) or by applying a weight inversely proportional to the contribution of the mode *m* to the total fluctuations (2b):
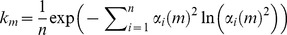
(2a)

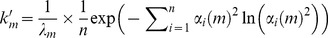
(2b)where *n* is the number of atoms in D, λ_m_ is the eigenvalue associated to mode *m* and 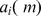 is the contribution of atom *i* to the domain D motion described by mode *m*:
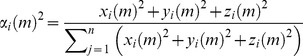
(3)where 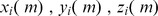
are the three components of atom *i* in mode *m* and *n* the number of atoms in D.

The contribution of a domain D to the global motion described by a mode *m*, expressed as a percentage, was calculated as:
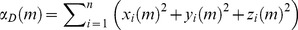
(4)


#### Analysis of intramolecular communications

Intramolecular communications in all studied mutants were built, analyzed and visualized with MONETA [41], using a more advanced version of this tool [63]. The principle of MONETA consists in building a modular network representation of the protein, composed of clusters of residues representing *independent dynamic segments* (*IDSs*) and of chains of residues representing *communication pathways* (*CPs*). The representation is derived from the topological description of the protein and from inter-residue dynamical correlations extracted from MD simulations. *IDSs* were identified by the *Local Feature Analysis* (LFA) [64]. This statistical approach is based on a transformation of global modes computed by Principal Component Analysis (PCA) into “local modes” describing local dynamical behavior, independent from the rest of the protein motions. PCA was performed for each model of KIT, on the Cα covariance matrices calculated on the last 30 ns of each simulation replica concatenated as one 60 ns simulation. MONETA analysis of communication pathways, as entirely based on dynamical correlations, is highly sensitive to the autocorrelation of the system [63]. In order to avoid or reduce the bias related to autocorrelations, we excluded the first 10–20 ns of each trajectory from consideration. From the 3N eigenvectors associated with 3N eigenvalues, the first 14, 21, 17, 15 and 16 eigenvectors were sufficient to describe 80–82% of the total Cα-atoms fluctuations of KIT^D816H^, KIT^D816Y^, KIT^D816N^, KIT^D816V^, KIT^V560G^ and KIT^V560D^ respectively. These vectors were consequently used to apply the LFA formalism as described in details in [41], [63]. To discriminate between correlated and uncorrelated residues, a threshold value P_cut_ was arbitrary chosen for each model in such way that about 1.0–1.2% of all LFA cross-correlations were above P_cut_. P_cut_ was 0.030 for KIT^D816H^, 0.033 for KIT^WT^, KIT^D816V^, KIT^V560G^ and KIT^V560D^, 0.040 for KIT^D816N^ and KIT^D816Y^. Distance matrices consisting of the average smallest distances between each residue pairs were computed using the g_mdmat program of GROMACS 4.5.3 [65]. Two residues *i* and *j* were considered as neighbors if the average smallest distance between them was lower than a given threshold d_cut_ of 3.6 Å. To characterize *communication pathways* we used the concept of communication propensity [66] as described in our previous work [41]. The *CPs* are grown in a way that ensures that any two adjacent residues are connected by non-covalent interactions and that every residue in the *CP* is connected to any other point by a short *commute time* (*CT*). Non-bonded interactions were recorded along the MD simulations using LIGPLOT [67]. Two residues were considered as interacting when they formed at least one non-bonded interaction for at least 50% of the simulation time. To discriminate between large and short *commute times*, a threshold *CT*_cut_ was chosen so that highly connected residues communicate efficiently with about 20% of the total number of residues in the protein, consistently with [66]. The threshold value was 0.09 for all studied models.

#### Statistical analysis and graphics

Statistical analysis was performed with the R software [68], [69], VMD 1.8.7 [70] and PyMOL 1.2 [71] were used for secondary structure assignment and visualization. The structure, interactions and communications in proteins were visualized with PyMOL and GEPHI [72] modules incorporated in MONETA [63].

## Results

KIT models bearing a gain-of-function mutation either in the A-loop (D816H/Y/N) or in the JMR (V560G/D) were generated from the crystallographic structure of the cytoplasmic region of KIT^WT^ in the auto-inhibited inactive state (PDB id: 1T45) [34]. For each mutant model, MD simulations were carried out (**Table S1**) in duplicates (replicas **1** and **2**) for 50 or 70 ns. The KIT mutant variants are referenced as KIT^D816H^, KIT^D816N^, KIT^D816Y^, KIT^V560G^ and KIT^V560D^. For an integral vision of mutation effects in KIT we took into consideration our previously published MD simulations of KIT^WT^ and KIT^D816V^ [40]. The analysis of these proteins was complemented and the related results were commented together with the newly obtained data to achieve a generic description of the hot-spots mutations effects on KIT.

### I. General observations

**Figure 3:**
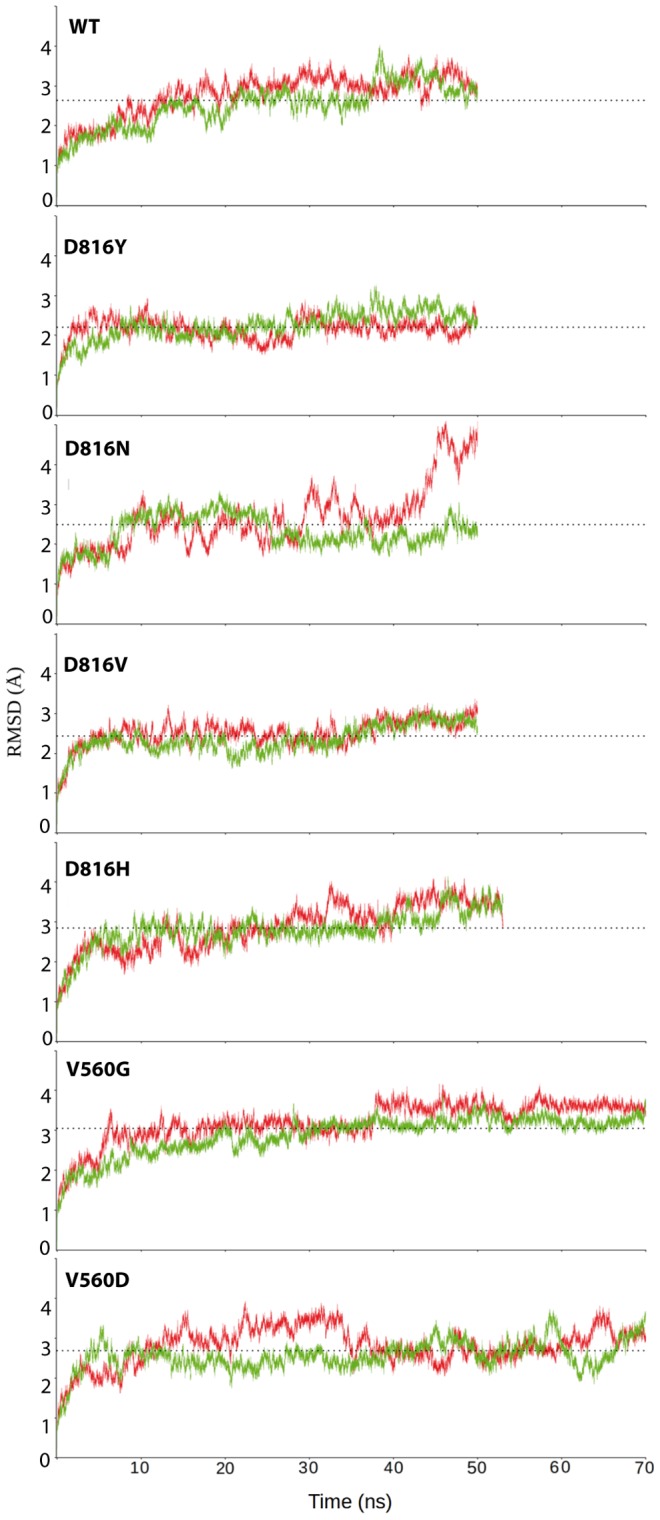
MD simulations of the KIT cytoplasmic domain in the inactive state. The RMSDs (in Å) per residue were calculated from trajectories 1 (red) and 2 (green) of MD simulations of KIT^D816Y^, KIT^D816N^, KIT^D816H^, KIT^V560G^ and KIT^V560G^. Horizontal doted lines indicate the RMSDs mean values.

The global dynamical behavior of each simulated system, KIT^D816H/Y/N^ and KIT^V560G/D^, was first characterized by Root Mean Square Deviations (RMSDs) from the initial coordinates computed on the backbone atoms (**Figure 3** and **Table S2**). This analysis evidenced that (i) for each protein, except KIT^D816N^, the RMSD profiles of the two simulation replica are very similar, indicating a good reproducibility of the MD simulations; (ii) a short relaxation period, 2-ns (KIT^D816Y^ and KIT^D816N^) or 5-ns (KIT^D816H^, KIT^V560G^ and KIT^V560D^), is required to achieve a reasonable stability of the systems; (iii) the average conformational drifts are in the range 2.16–3.22 (±0.26–0.79) Å, in good agreement with the values obtained previously for KIT^WT^ and KIT^D816V^; (iv) a reasonable RMSD convergence is observed at the end of the simulations of either 50 ns (KIT^D816N^, KIT^D816H^ and KIT^D816Y^) or 70 ns (KIT^V560D^ and KIT^V560G^). The RMSD profiles of the two MD simulations of KIT^D816N^ are dissimilar: the 1^st^ replica displays highly increased deviations after 30 ns, not observed in the 2^nd^ trajectory. This increase arises from the higher flexibility of the few residues at the C-terminal extremity of the model. The RMSD profiles computed on the backbone atoms of KIT^D816N^ excluding these residues are similar for the two replicas (**Figure S1**). The RMSD profiles computed on different structural parts of the protein evidenced that the conformational drifts of the JMR are significantly larger than those of the other KIT regions in all analyzed proteins (**Figure S2**).

The conformational stability of the studied systems was further investigated through a convergence analysis of the trajectories. This analysis uses a set of *reference* structures and a RMSD cutoff for clustering the MD conformational ensemble from a trajectory in *reference* groups (**Materials and Methods**). The population of each cluster is analyzed in terms of conformations issued from either the first or the second half of the trajectory. A RMSD cutoff value of 2.5 Å leads to the selection of 2–7 *reference* structures in KIT^D816N^, KIT^D816Y^, KIT^D816V^ and KIT^WT^, and many more (5–10 *reference* structures) in KIT^D816H^ and KIT^V560D^ (**Table S3**). The increased number of *reference* structures indicates a larger conformational exploration by the KIT^D816H^ and KIT^V560D^ compared to KIT^WT^ and other mutants. As an increased number of clusters diminishes their chance to be populated by both halves of the trajectory, we repeated the convergence analysis with a slightly higher cutoff of 3 Å for KIT^D816H^ and KIT^V560D^, which led to the identification of 2–6 *references*. Convergence of the simulations, assessed by the convergence criterion *c*, was improved in all studied mutants compared to KIT^WT^ (**Table S3**). In particular, KIT^D816Y^, KIT^D816H^ and KIT^V560D^ display a very high convergence quality (*c*≥0.8 *vs* 0.4–0.5 in KIT^WT^) in the two simulation replicas.

**Figure 4:**
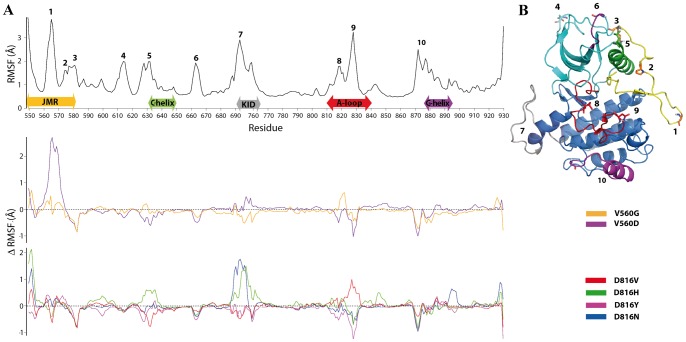
Displacements and fluctuations of residues in KIT mutants. (**A**) The RMSFs computed on the carbon and nitrogen backbone atoms over the total production simulation time of KIT mutants were compared to those in KIT^WT^. (*Top*) RMSFs (Å) of KIT^WT^ [40] (graph in black) and the RMSFs difference (Δ RMSF, Å) between KIT^WT^ and mutants: (*Middle*) KIT^V560G^ (orange), KIT^V560D^ (purple), (*bottom*) KIT^D816V^ (red), KIT^D816H^ (green), KIT^D816Y^ (magenta) and KIT^D816N^ (blue). (**B**) The regions with significant average Δ RMSF values are displayed with different colors: JMR (yellow), A-loop (red), N- and C-lobe (cyan and blue) and KID (gray). The radii of the KIT cartoon representation is scaled relative to the RMSF values in KIT^WT^.

The protein flexibility was estimated by the Root Mean Square Fluctuations (RMSFs) computed for the C- and N-backbone atoms over the production time of each simulation. The RMSF mean values, averaged over the whole structure, are comparable between the different models, despite the distinct production times (48-ns or 65-ns): they range from 1.05 to 1.31 Å for KIT^D816H/Y/N^ and from 1.07 to 1.24 Å for KIT^V560G/D^. In all models, the most fluctuating regions are the JM-Switch (residues 560–570), the N-extremity of the C-helix (residues 631–634) and the preceding highly flexible loop (N-C-loop, residue 626–630), the anti-parallel β-sheet of the A-loop (residues 825–831) composed of two strands (β8 and β9), further referenced as the β-hairpin of the A-loop [73], and a highly solvent accessible coil region upstream the G-helix in the C-lobe (G-loop, residues 877–885) (**Figure 4** and **Figure S3**). The JM-Switch appears more flexible in KIT^V560G^ and much more flexible in KIT^V560D^ compared to KIT^WT^ and the other mutants. Its maximal RMSF value is increased from 3.8 (KIT^WT^) to 6.5 Å (KIT^V560D^).

RMSD and convergence analysis enabled validation of the produced MD trajectories and assessment of their reproducibility. Together with RMSF analysis they show that the studied mutations influence the dynamical behavior of KIT, globally inducing a stabilizing effect, manifested by smaller conformational drifts of the whole protein and more converged conformational ensembles in all mutants.

### II. Structural features of KIT^D816H/Y/N/V^ and KIT^V560G/D^

**Figure 5:**
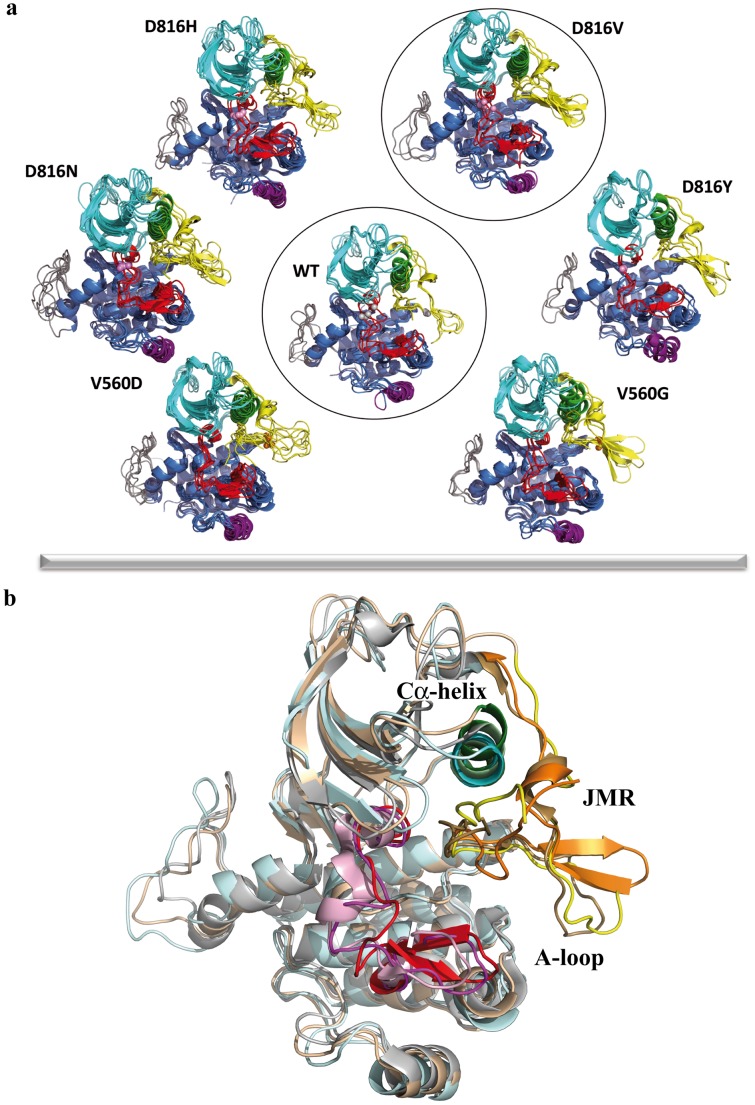
MD conformations of KIT cytoplasmic region in the native protein and its mutants. (**A**) Superposed conformations were selected by RMSDs clustering. Ribbon diagrams display the proteins regions or fragments with different colors: JMR (yellow), A-loop (red), N- and C-lobe (cyan and blue), C-helix in the N-lobe (green), G-helix in C-lobe (purple) and KID (gray). The point mutations in positions 816 and 560 are shown as balls. The earlier reported KIT^WT^ and mutant KIT^D816V^ are encircled. (B) Superimposed structures of the most representative MD conformations in the native KIT (I) (in wheat), its mutant D816H (II) (in pale cyan) and the crystallographic structure 1T45 (III) (in gray). Ribbon diagrams display the proteins regions or fragments with different colors: (I) JMR in yellow, Cα-helix in green, A-loop in magenta; (II) JMR in orange, Cα-helix in cyan, A-loop in red; (III) JMR in sand, Cα-helix in pale green, A-loop in pink.

Visualization and analysis of the clustered MD conformations of each simulated model show that (i) the general folding of KIT cytoplasmic region is conserved and (ii) two main regulatory fragments, the JMR and the A-loop, are predominantly sensitive to amino acid substitution at positions 816 and 560 (**Figure 5 a**).

#### Structural effects of the A-loop mutations in position 816

Previously we have shown that the D816V mutation induces a local destabilization of the short 3_10_-helix at residues 817–819 [40]. A similar effect of mutation D816H is evidenced when comparing the crystallographic structures of KIT^WT^ and KIT^D816H^ bound to the inhibitor sunitinib (PDB ids: 3G0E and 3G0F [30]) and was also observed in a recently reported *in silico* study [74]. Inspection of the clustered MD conformations from our simulations of KIT^D816H/Y/N^ again put in evidence complete or partial unfolding of the adjacent 817–819 3_10_-helix in A-loop (**Figure 5 a, b**). Considering the perfect agreement between these results, previous *in silico* studies and available experimental data, we interpret the structural modification in this region as principally induced by mutation in position 816.

**Figure 6:**
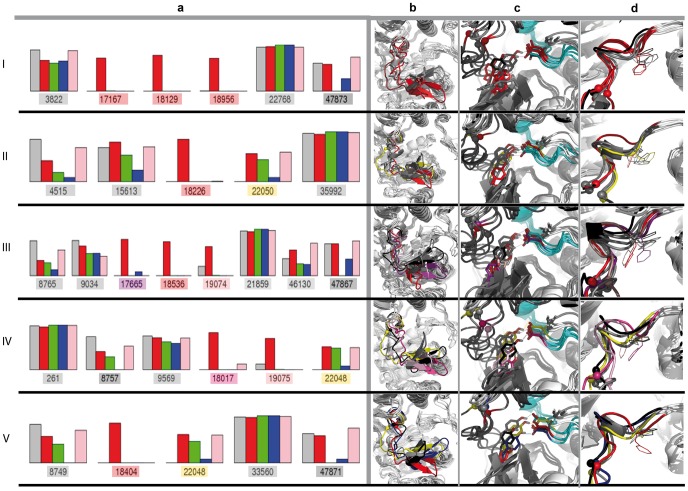
A-loop conformations in KIT^WT^ and KIT^D816H/V/Y/N^ mutants. (**a**) Cluster analysis of the MD conformations picked every 10 ps over the 96 ns of productive MD simulations were performed on the RMSDs computed for only the A-loop backbone atoms. Five different sets were produced through 5 independent runs of clustering (I–V). The composition of clusters is represented as colored barplots in a logarithmic scale: KIT^WT^ (gray), KIT^D816V^ (red) KIT^D816H^ (green), KIT^D816Y^ (blue), KIT^D816N^ (pink). The rank in the concatenated trajectory of the corresponding *reference structure* is reported in the bottom of each barplot and colored accoring to the population of the group (see below). (**b–d**). Superposition of the *reference structures* in each cluster analysis run. The *reference structures* are drawn as cartoons illustrating the A-loop conformation (**b**), the relative orientation of the residues Y823 (A-loop) and D792 (C-loop) (**c**) and the conformation of DFG motif (A-loop) (**d**). The A-loop is shown as cartoon diagrams, residues Y823 and D792 as sticks, F811 as thick lines; D/H/V/Y/N816 as sphere. The H-bonds are shown by dotted lines. The A-loop conformations observed in all simulated proteins including KIT^WT^ are shown in grey; conformations detected in all protein but one mutant are distinguished in black or blue; conformations detected only in the mutants are in yellow; the proper conformations of KIT^D816V^ mutant are in red; characteristic of the pair of mutants, KIT^D816V/Y^ or KIT^D816V/N^ are in purple or in pink.

To further explore the conformational variability of the A-loop over the MD trajectories of KIT in the native and A-loop-mutated forms, we performed clustering of the A-loop conformations (**Materials and Methods**). The A-loop conformations in KIT^D816V^ display a significantly increased variability with respect to the other models (**Figure 6a**, red bars). Some clusters are represented in all models including KIT^WT^ (**Figure 6**, **b–d**, grey cartoons), others are characteristic of the four mutants KIT^D816V/H/Y/N^ (in yellow) or of two mutants, either KIT^D816V/Y^ (in purple) or KIT^D816V/N^ (in magenta), or of only KIT^D816V^ (in red). The β-hairpin of the A-loop adopts either typical conformations widely distributed (in grey/yellow) or alternative conformations detected in only one or two mutants (in red/pink) (**Figure 6 b**). In the alternative conformations, the β-hairpin is shifted away from the active site impairing H-bonding between D792 and Y823 (**Figure 6 c**). In conformations specific to one or two mutants, the backbone of the DFG motif and the side chain of F811 are displaced from the ATP-binding site, a feature characterizing the “in” conformation and residues 816–823 adopt a mainly coiled and elongated conformation (**Figure 6 b**).

**Figure 7:**
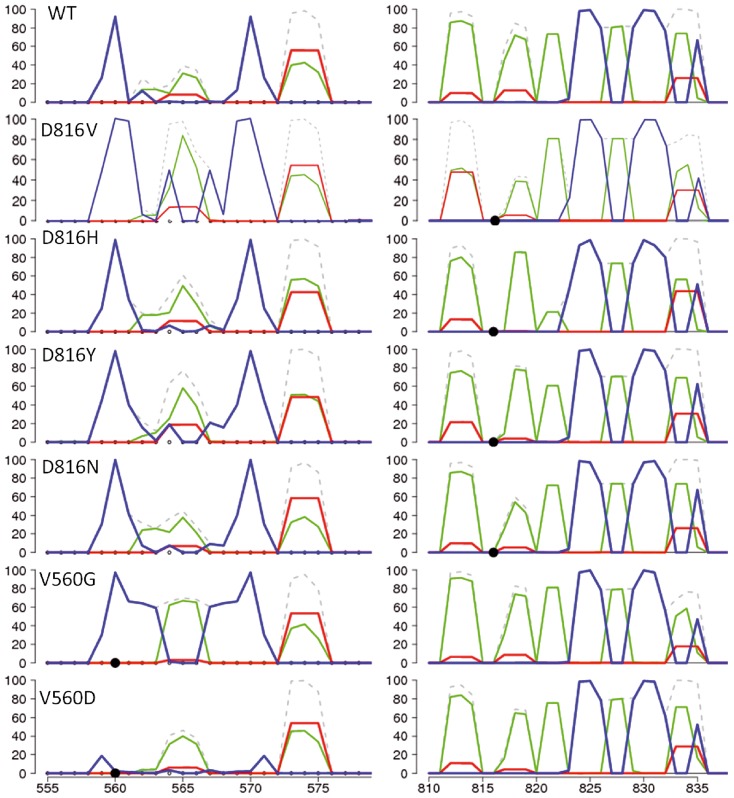
Variable of secondary structures in the cytoplasmic region of KIT^WT^ and mutants, KIT^D816H/Y/N^ and KIT^V560G/D^. Secondary structure assignments for the JMR (left) and the A-loop (right) were averaged over the two replica of each MD simulations (2×48 or 2×65 ns) of KIT^WT^ and mutants. For each residue, the proportion of each secondary structure type is given as a percentage of the total simulation time and shown with lines of different colors: 3_10_-helices (red), antiparallel β-sheet (blue), turns (green), total structure (dashed gray).

To quantify the structural alteration near the mutation site, we summed the occurrences of each secondary structure element at each position of the A-loop upon the productive simulation time (2×48 ns) for each KIT^D816V/Y/N/H^ model. This statistical characterization indicated that the appearance of a 3_10_-helix at residues 817–819 is diminished by a factor of 3 in KIT^D816V/Y/N^ and by a factor of 13 in KIT^D816H^ with respect to KIT^WT^ (**Figures 7** **and** **8**). Monitoring the H-bonds pattern in this region enabled to correlate the 3_10_-helix status from partial to complete disappearance with a systematic weakening of the local H-bond network in the A-loop of the mutants (**Table S4**). The number of occurrences of a turn formed by residues 820–823 is also diminished in all mutants compared to the KIT^WT^. Moreover the distance between the Cα atoms of residues 816 and 823 is increased in all studied mutants (11.5–12.5 Å) with respect to KIT^WT^ (10.5 Å), indicating an elongation of this region upon mutation.

**Figure 8:**
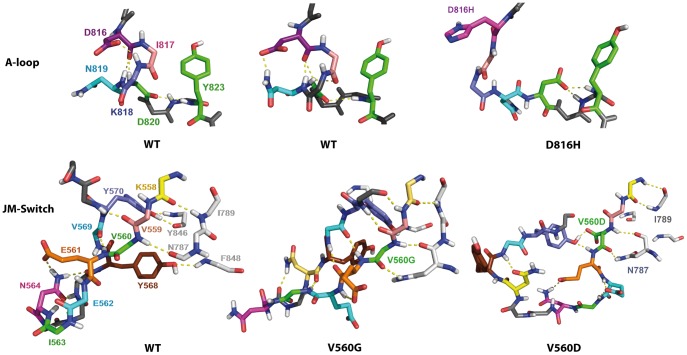
H-bond patterns in the A-loop and the JMR. *Top*: H-bonds stabilizing the small 3_10_-helix in the A-loop of KIT^WT^ (in two different projections) and coiled structure of the A-loop observed in mutant KIT^D816H^. *Bottom*: H-bonds of the JMR and the N-lobe residues stabilizing the coiled structure of the JM-Switch in KIT^WT^ and KIT^V560D^ and nearly antiparallel β-sheet in KIT^V560G^. All residues presented as sticks, each residue is labeled in KIT^WT^ and specified by color retained for the same residue in the mutants, only the side chains participating in the H-bonds are shown. The H-bonds are shown as dotted lines.

These analyses demonstrate a local effect induced by D816H/Y/N/V mutation on the A-loop structure, manifested as a destabilization of the transient 3_10_-helix at residues 817–819 and of the turn structure formed by residues 820–823. The magnitude of these short-range effects is variable in the different studied mutants, and is particularly noticeable in KIT^D816V/H^.

Beyond the impact on the A-loop, comparison of the superimposed representative MD conformations selected by clustering also indicates an influence of D816H/Y/N mutation on the distant JMR, manifested by an increased β-strand folding of the JM-Switch (residues 560–570) (**Figures 5** and **7**) as previously observed in KIT^D816V^ [40]. The JM-Switch exhibits a well-folded anti-parallel β-sheet in 30–45% of the representative MD conformations in KIT^D816H/Y/N^. In KIT^WT^ the JM-Switch has a mainly coiled structure, while it adopts a perfectly folded β-sheet structure in 97% of dynamic conformations of KIT^D816V^ conformations. Consequently, KIT^D816H/Y/N^ display a conformational state intermediate between those observed in KIT^WT^ and KIT^D816V^. In addition, the occurrences of a β-sheet folded conformation in the mutants are correlated with the length of the β-strands: from 1–3 amino acids (aas) in KIT^WT^ up to 4 aas in KIT^D816H^ and 5 aas per strand in KIT^D816V/Y/N^. Using two kinds of algorithms designed to predict secondary structure elements, without alignment prior to the predictions (PREDATOR and GOR4) or based on multiple alignment (SORMA, SIMPA96, PHD and Jnet), we explored the proper folding of the 20 amino acids sequence of the JMR (residues 555–575). Predictions indicate a relatively high probability of either β-strands or α-helices in the segment 555–562 and β-strands in the segment 568–571, with a random coil of 5 residues linking them. The predicted β-strands correspond well to the folded structure of the JM-Switch (residues 560–570) observed in the MD conformations of the mutants KIT^D816V/H/N/Y^.

The JM-Switch position relatively to the kinase domain (KD) was monitored by (i) the distance D_JMS_ between the JM-Switch and the C-lobe, and (ii) the angle A_JMS_ formed by the centers of mass of the JM-Switch, the KD and the C-lobe (**Materials and Methods**). The mean values of D_JMS_ and A_JMS_ increased from 12.0 Å and 75.8° in KIT^WT^ to 12.6–13.3 Å and 77.1–79.7° in KIT^D816V/H/Y/N^ with maximal values (18.5 Å and 91.6°) reached in KIT^D816V^ (**Table S5**). Both metrics demonstrate that the JM-Switch is more distant from the KD in the mutants than in KIT^WT^. Consequently, mutation D816H/Y/N/V induces (i) a diminished attachment of the JM-Switch to the kinase domain respectively to KIT^WT^ and (ii) stabilization of the JM-Switch β-sheet structure.

#### Structural effects of the JMR mutations in position 560

Analysis of the representative MD conformations evidenced a striking structural dissimilarity of the JMR in KIT^V560G/D^ mutants. Principally, the JM-Switch displays a large and highly stable anti-parallel β-sheet in KIT^V560G^, whereas this fragment is completely unfolded in KIT^V560D^ (Figures 5 and 7). Moreover, the position of the JM-Switch respectively to the KD is variable in both mutants. D_JMS_ distance and A_JMS_ angle values in KIT^V560G^ (12.9 Å and 76.5°) are only slightly increased with respect to those observed in KIT^WT^ (12.0 Å and 75.8°), while their values in KIT^V560D^ reach up to 17.2 Å and 87.2° respectively (Table S5). These results indicate that the position of a twisted hairpin of the JMR in KIT^V560G/D^ mutants differs either slightly (KIT^V560G^) or significantly (KIT^V560D^) from its position packed to the C-lobe in KIT^WT^. Apparently, the substitution of a valine (V) in position 560 to a negatively charged aspartate (D) or to a tiny uncharged glycine (G) provokes an important structural impact on the JM-Switch, a fragment adjacent to the point mutation V560G/D. This impact is manifested in both mutants as a displacement of the JM-Switch towards an axial position respectively to the kinase domain, which in KIT^V560G^ is accompanied by a considerable JM-Switch folding. It is worth noting at this point that both kinds of substitution in position 560 seem to have no structural effect on the distant A-loop.

To interpret the observed structural effects of V560G/D mutations, we considered the hydrophobicity of residues according to a scale ranging from 0 (glycine, neutral) to 100 (phenylalanine, highly hydrophobic) [75]. On the one hand, the scores of 76 for valine (V) and 55 for aspartate (D) may partially explain the increased solvent accessibility of the JM-Switch upon V560D mutation. The valine substitution by the easily solubilised aspartate may be a major factor contributing to the emergence of a great variability of randomly coiled and solvent-accessible conformations of the JM-Switch. In addition, it was suggested that the H-bonds formed by the carboxylic acid of an aspartate with its environment can compete with structural backbone-backbone H-bonding, and this competition tends to destabilize β-sheets. Aspartate thus plays the role of a “beta breaker” [75]. On the other hand, although the small non-polar amino acid G is usually considered as a “structures breaker”, it may contribute to a stabilization of β-sheets if it is paired with an aromatic residue [76]. We observed that in the MD conformations of KIT^V560G^, G560 is paired with Y570 and the aromatic cycle of tyrosine stands over the glycine, stabilizing the position of two β-strands at a closer distance to each other than in the MD conformations of KIT^WT^ (**Figure 8**).

In KIT^WT^, the JMR binds to the C-lobe of the kinase domain through the stable (99%) backbone-backbone H-bonds V560•••N787 and K558•••I789 (**Figure 8**). The occupancy of these H-bonds in KIT^V560D^ and KIT^V560G^ is reduced by 15% and 30% respectively. Moreover the mixed side chain-backbone H-bonds Y568•••F848 and Y570•••Y846 contributing to the JMR binding to the KD in KIT^WT^ are significantly diminished or completely disappeared in KIT^V560G/D^. In contrast, the intra-JMR H-bond patterns, very limited and consisting of rare contacts (*i.e.*, V570•••V559 and V569•••E561) in KIT^WT^, appear as composed of multiple, well-ordered, short and stable backbone-backbone contacts in KIT^V560G^, stabilizing a near anti-parallel arrangement of the two β-stands of the JM-Switch. Thus, mutation V560G contributes to (i) weakening the JMR attachment to the kinase domain and (ii) stabilization of interactions that favor the JM-Switch folding. In KIT^V560D^ the intra-JMR contacts are limited to several mixed or side chains contacts, randomly distributed in the JM-Switch. Consequently, mutation V560D induces (i) a diminished affinity of the JM-Switch to the kinase domain respectively to KIT^WT^ and (ii) destabilization of the JM-Switch β-sheet structure. It should be stressed out that some residues involved in the JMR binding to the KD in KIT^WT^ play crucial functional roles. Particularly, Y568 is very important for negative regulation of the receptor as it has been shown to be an interaction site for negative regulators [77].

### III. Effects induced by mutations D816H/Y/N/V and V560G/D on KIT internal dynamics

The global motions in KIT mutants were further analyzed by Principal Component Analysis (PCA) and compared to those in the native protein. Earlier we showed that the D816V mutation alters the global conformational dynamics of KIT, in particular by the disruption of an allosteric coupling between JMR and kinase domain [41]. In the present study we applied PCA to all studied KIT mutants with the aims of complementing the results obtained for KIT^D816V^ and distinguishing the effects produced by the different mutations on (i) the collective motions of KIT functional regions and (ii) the allosteric coupling between the spatially distant regulatory fragments, A-loop and JMR, established in KIT^WT^.

**Figure 9:**
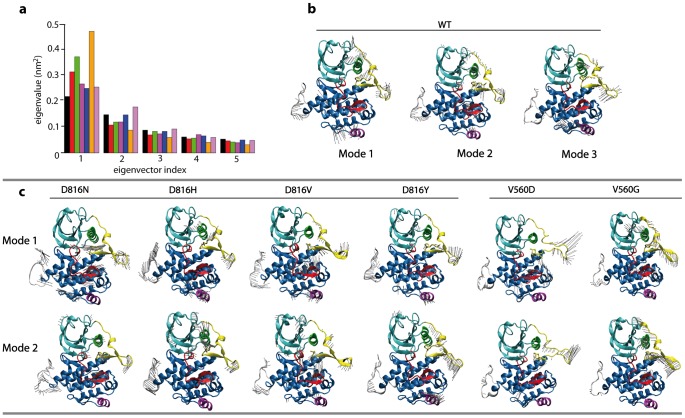
Principal Component Analysis of KIT cytoplasmic region in the inactive state. Calculation was performed on the backbone atoms of KIT^WT^ and mutants, KIT^D816H/Y/N^ and KIT^V560G/D^, combining the MD trajectories 1 and 2 (total of 96-ns production simulation time for KIT^D816H/Y/N^ mutants, and 130-ns for KIT^V560G/D^ mutants) and taking the average MD conformations as references for the RMS fits. (**a**) A diagram gives the eigenvalues spectra of KIT^WT^ (black) and mutants, KIT^D816V^ (red), KIT^D816H^ (green), KIT^D816Y^ (purple), KIT^D816N^ (blue), KIT^V560G^ (orange) and KIT^V560D^ (mauve), in descending order. Atomic components in modes 1–3 for KIT^WT^ (**b**) and modes 1–2 for KIT mutants (**c**) are drawn as dark grey arrows on the protein cartoon representation. Some regions or fragments of the proteins are displayed with different colors: JMR (yellow), A-loop (red), N- and C-lobe (cyan and blue), the C-helix in the N-terminal (green), the C-terminal helix (purple) and KID (gray).

In all studied mutants of KIT, the number of significant modes is reduced respectively to KIT^WT^. The increased contribution of the 1^st^ mode in all mutants compared to KIT^WT^ (**Figure 9 a**) clearly indicates a simplification of motions in the mutants compared to the native protein. Therefore three modes for KIT^WT^ and two modes for KIT mutants were used to illustrate and compare KIT motions. In all models, the residues undergoing the main motions are localized in the JMR, in the N-G-loop (residues 864–876), the N-C-helix and N-C-loop (residues 631–634 and 626–630) and in the β-hairpin of the A-loop (residues 825–830). All these fragments have been reported as essential for activation/deactivation mechanisms of KIT [7].

The 1^st^ PCA mode of KIT^WT^ evidences large correlated motions of nearly the whole JMR, the N-G-loop, the flexible and solvent-accessible loop linking β-4 and β-5 strands in the N-lobe (“β4-β5-loop”, residue 660–667), the β-hairpin of the A-loop, and the N-terminal part of the C-loop (**Figure 9 b**). The 2^nd^ and 3^rd^ modes denote motions in the same regions with additional movements in the central part of the A-loop (residues 816–823), and either in the P-loop (2^nd^ mode) or in the DFG motif of the A-loop (3^rd^ mode).

The two first PCA modes of KIT^D816H/Y/N/V^ mutants depict correlated motions of globally the same segments as in KIT^WT^ (**Figure 9 b, c**). In all mutants, the 1^st^ mode covers fewer motions in the N-lobe than in KIT^WT^, and depicts essentially motions of the JM-Switch (KIT^D816H/V/Y^), and in the A-loop (KIT^D816V/N^). In each mutant, highly concerted motions of the JM-Switch are represented either by the 1^st^ mode (KIT^D816V^), or the 2^nd^ mode (KIT^D816Y/N^) or both 1^st^ and 2^nd^ modes (KIT^D816H^). The two first PCA modes of KIT^V560D^ reveal mainly motions of the whole JMR. These motions are strongly concerted and characterized by a particularly high amplitude, coherent with the strongly increased atomic fluctuations in this region evidenced by RMSFs. The first PCA mode of KIT^V560D^ depicts essentially high-amplitude motions of the JM-Switch, which is consistent with a strongly increased flexibility of this segment in KIT^V560D^ compared to KIT^WT^ (**Figure 9 b, c**). The motions of the JM-Binder (residues 553–559), JM-Switch (residues 560–570) and JM-Zipper (residues 571–581) appear extremely concerted. These JMR motions correlate with few or no motions in the kinase domain. The highly collective and concerted motions of KIT^V560G^ show that the JMR motions correlate with motions in the C-helix and A-loop in both modes. Visualization of the first modes in each model suggests the presence of more collective motions of the JM-Switch in the mutants compared to the native KIT, consistent with what was evidenced in KIT^D816V^. This finding was quantitatively assessed by computation of JM-Switch motions collectivity in the first 5 modes of each model, either in absolute values (**Figure S4 a**) or weighted by the contribution of each mode to the global motion of the model (**Figure S4 b–c**).

To compare quantitatively the first modes in the different KIT proteins, we computed the resultant of the JM-Switch atomic motions. Increased amplitude and enhanced contribution of the JM-Switch motions to the first modes are observed in all mutants, except for KIT^D816N^, respectively to KIT^WT^ (data not shown). The first modes of KIT^D816N/H^ reveal essential motions of the *pseudo*-KID, which was then discarded to avoid the bias induced by this engineered fragment. The re-computed norms evidenced an increase in the amplitude of JM-Switch motions in all mutants compared to KIT^WT^. Consequently, the PCA analysis indicates a reduced diversity of collective motions together with amplification of some essential predominant motions in the dynamics of the KIT mutants compared to KIT^WT^. In all studied mutants the degree of collectivity of the JM-Switch motions is enhanced.

### IV. Inter-domain communication in KIT

We evidenced that the local structural effect induced by each gain-of-function point mutation, D816H/N/Y/V or V560D/G, corresponds to alteration of the H-bonds pattern involving the residues neighboring the mutation site. To understand the origin of the observed long-range structural effects induced by the point mutation D816H/N/Y/V localized in the A-loop on the distant JMR, we characterized the intra-protein communication pathways in all studied models, looking for interaction networks linking these fragments in particular.

We recently developed a novel method called the MOdular NETwork Analysis (MONETA), designed to characterize communication pathways inside a protein [41], [63]. MONETA explores the intramolecular non-bonded interactions and the inter-residues dynamical correlations computed from MD trajectories to identify, on the one hand, clusters of locally coupled residues and, on the other hand, chains of non-covalently connected residues displaying concerted motions at long range.

Such approach applied to KIT put in evidence a well-established communication between the JMR and the A-loop, through the catalytic loop [41]. D792 and Y823, linked in KIT^WT^ by a strong and stable H-bond, were identified as key residues in establishing communication pathways. An absence of this H-bond in KIT^D816V^ revealed the disruption of the allosteric communication between the JMR and the A-loop is likely associated to the mutation-induced structural and dynamical effects. These outcomes prompted us to modulate the communication in KIT by *in silico* mutagenesis. A second mutation D792E, predicted by MONETA, was introduced in KIT D816V mutant, which enabled to neutralize the long-range effect of D816V and restore the communication profile observed in the native protein [41]. This observation confirmed our hypothesis of an inherent link between communication, dynamical behavior and structural features of the regulatory fragments of KIT.

Each studied KIT mutant was analyzed by MONETA with the aim of examining their communication profiles and relating them with the structural alterations induced in KIT by the mutations. By using the most advanced version of MONETA [63] and a large and diverse MD simulations data set, we gained comprehensive insight into the mechanisms of allosteric transitions in KIT.

#### Identification of the independent dynamics segments

As a first step of such characterization, the regions of KIT representing the most striking local features of the protein internal dynamics were identified in each analyzed mutant by a statistical technique known as *Local Feature Analysis* (LFA) [64], adapted to study essential dynamics in proteins [78]. The LFA formalism enables to select *seed* residues, representing the most striking features of the local dynamics of the studied system, and to define clusters formed around each *seed* by neighboring residues displaying concerted local atomic fluctuations. These clusters are named *Independent Dynamic Segments* (*IDSs*), as they represent regions of the protein that display independent dynamical behavior [41], [79].

In KIT mutants the number of modes used for LFA varied from 14 (KIT^D816H^) to 21 (KIT^D816Y^). The number of *IDSs* defined around all *seed* residues in the analyzed KIT models, varies from 6 to 8 in KIT D816^H/N/Y^ and KIT V560^D/G^, while in KIT^WT^ and KIT^D816V^ it reaches up to 10. The sum of all *IDSs* comprises from 48 (KIT^V560D^) to 110 (KIT^D816V^) residues which represents from 15 to 33% of KIT protein.

**Figure 10:**
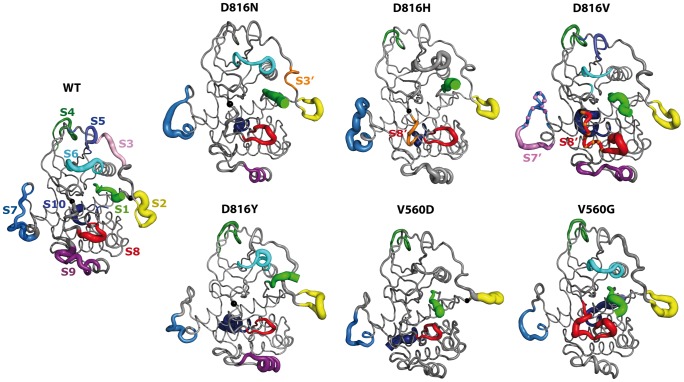
Independent dynamic segments identified in KIT cytoplasmic region. Structural mapping of the *Independent Dynamic Segments* (*IDSs*) identified in KIT^WT^ and KITmutants. The average conformations are presented as tubes. The size of the tube is proportional to the by-residue atomic fluctuations computed on the backbone atoms. *IDSs* are referred to as 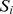, where i = 1, 2…10, labeled in KIT^WT^ and specified by color retained for the *IDSs* in mutants. The largely modified or newly found *IDSs* in the mutants are referred to as 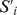.

For a comparative analysis of the *IDSs* in all models they are referred to as S_i_, where i = 1, 2…10. In KIT^WT^, further used as a reference, the 10 identified *IDSs* (S1, S2, …S10) are located in the N- and C-terminal extremities (S1 and S10), the JM-Switch (S2), the JM-Zipper (S3), the *pseudo*-KID (S7), two solvent exposed loops in the N-lobe (S4 and S5), the N-terminal part of C-helix and the N-C-loop (S6), the solvent-exposed G-helix and N-G-loop in the C-lobe (S10), and the A-loop (S8) (**Figure 10**). These regions were identified as the principal components of the regulation of kinase activity [41].

In KIT mutants the *IDSs* are overall located in the same regions as in KIT^WT^. The hotspot mutational residues 560 and 816 are either directly involved in an *IDS* (V560 belongs to S2 in the JM-Switch) or adjacent to a residue participating in an *IDS* (Y823 belongs to S8 in the A-loop) in KIT^WT^ as well as in the mutants. However, many *IDSs* in the mutants show modification either in terms of size (number of residues) or of position in the sequence. In the N-lobe, S4 is lacking in KIT^D816N^ and S5 is absent in all mutants, except KIT^D816V^. The *IDS* S6 is slightly shifted in KIT^D816V^ and extended in KIT^D816Y^, KIT^D816N^ and KIT^V560G^, whereas it is absent in KIT^D816H^ and KIT^V560D^ (**Figure 10**). The disappearance of S4, S5, S6 and S9 in several mutants correlates well with the more collective motions of these mutants. For example, KIT^D816H^ displays (i) a reduction of the proportion of residues in *IDSs* and of the number of *IDSs* and (ii) largely more collective motions compared to the KIT^WT^. As *IDSs* are defined from PCA, it is expected that they reflect in some way PCA results. More collective motions mean less striking local features.

The largely modified or newly found *IDSs* in the mutants respectively to KIT^WT^ are referred to as 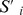. Particularly, S3 (residues 576–582 in KIT^WT^) is considerably shifted in KIT^D816N^ (S3′, residues 572–575) towards S2 (JM-Switch) and is absent in the other mutants. The *IDS* S8 in the A-loop (JM-Zipper, residues 824–831 in KIT^WT^) is significantly expanded in KIT^D816V^ towards residue V816, up to the position 817 (S8′). Moreover, in this mutant two *IDSs* are superimposed at residues 817–823 to form a duplicated *IDS*, S8 and S8′. In KIT^D816H^ two *IDSs* in the A-loop, S8′ (residues 817–822) and S8 (residues 824–831), are separated by a unique residue Y823. Such expansion and/or duplication of the A-loop *IDSs* is a consequence of increased concerted local motions in the N-terminal part of the A-loop in the D816V/H mutants compared to KIT^WT^ (**Figure 9**). This *IDS* S8 is extended in KIT^V560G^ to residues 816–831, consistent with the increased RMSFs of the entire A-loop in this mutant.

It is worth noting that the four *IDSs* – S3 (JM-Zipper), S4 and S5 (two solvent exposed loops in the N-lobe) and S6 (N-terminal part of C-helix and N-C-loop) – are observed simultaneously only in KIT^WT^. Such observation indicates a more correlated dynamical behavior of the structurally neighboring N-lobe and JM-Zipper in the mutants. The role of an intrinsic disorder of this region in the inactive state of the RTK EGFR was described [80]. Particularly, it was evidenced that a diminished disorder upon activation of the receptor favors dimerization of the intracellular domain. Comparative study of the native EGFR and its mutants has been used by the authors to demonstrate a stabilization of the N-lobe and the JM-Zipper in some mutants, which lead to the oncogenic activation of these mutants.

#### Communication pathways

As a second step in the analysis of communications between spatially distant regions in KIT we computed for each model the total number of *communication pathways* (*CPs*), the number of highly connected residues (“*hubs*”), the number of short *commute time* (*CTs*) per-residue, the length and the extension of the *CPs*. A *CP* is considered as short, intermediate or long if it involves respectively 2–3, 4–5 or more than 5 residues. A *CP* is considered as local or extended if the first and the last residues of this *CP* are distant by less or more than 10 Å respectively.

**Figure 11:**
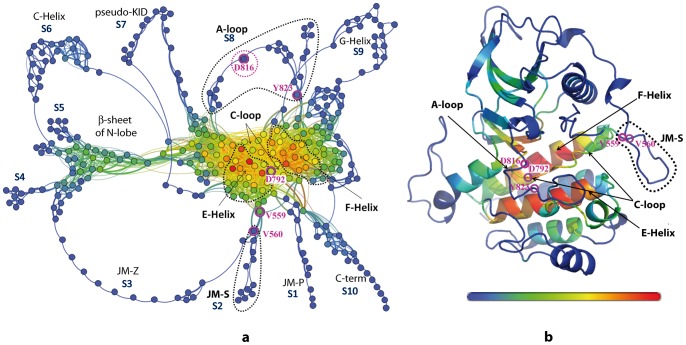
Communication pathways of cytoplasmic region in KIT^WT^. (**a**) 2D graph of a global topology the inter-residues communications. Residues are represented by points, c*ommunication pathways* (*CPs*) are depicted by bold lines and two connected residues by a thin line. Structural fragments involved in *Independent Dynamic Segments* (*IDSs*), are labelled as S_i_ where i = 1, 2, …10. (**b**) 3D structural mapping of the communication efficiently of KIT residues. The average MD conformation is presented as cartoon. Residues in 2D (**a**) and 3D (**b**) representations are coloured from blue through green and yellow to red according to their communication efficiency, estimated as the number of residues to which they are connected by at least one *CP*. Residues V560, V559, D792, D816 and Y813 are encircled by magenta. Clusters corresponding to protein segments of interest are contoured by dotted lines. 2D and 3D graphs and drawn with Gephi and PyMOL modules incorporated in MONETA.

The general landscape of *communication pathways* and the mapping of communication efficiency of residues in KIT^WT^ is illustrated as two- and three dimensional graphs (**Figure 11**). As we pointed earlier, *IDSs* in KIT are essentially composed of residues displaying fast communications only with neighboring residues along the protein sequence (1D) and in the 3D structure, and minimally coupled to the other regions of the protein [41]. Consequently, there is only one or very few *CPs* generated by residues involved in *IDSs*. All *IDSs* are connected by their extremities in the global *communication network* (*ComNet*) of the native protein by means of 2 404 pathways of different lengths.

We found that 44 residues of KIT^WT^ communicate fast with more than 10% of the protein residues and were identified as highly communicating residues or “*hubs*”. The maximal number of *CPs* reaches up to 67 per residue. The majority of the highly communicating residues is localized in the E- and F-helices, which contain nearly all *“hubs”* grouped in two very dense clusters of *CPs* (**Figure 11** **a**). These well-defined clusters are separated by a groove characterizing the *CPs* formed by residues from the C-loop. These residues together with those composing the short helices in the C-lobe and the β-sheet of the N-lobe communicate moderately, forming secondary communication clusters. The central dense clusters and the secondary clusters are interconnected through the lowly communicating regions, the hinge and the C-terminal loop of the C-helix. These two structural fragments are the vital communication channels between the N- and C-lobes.

Main regulatory fragments of KIT, A-loop and JMR, are composed of poorly communicating residues and are connected by their two ends, either directly or through the secondary clusters, to the highly communicating regions. The other protein fragments involved in the *IDSs* are the solvent-exposed coils or the solvent-exposed parts of helices (*i.e*., the C- and G-helices) and, like the A-loop and the JMR, they show a very limited number of *CPs*. The JMR containing three *IDSs* (S1, S2 and S3) formed by residues from the JM-Proximal, the JM-Switch and the JM-Zipper respectively, has three different sets of communication pathways interconnected through residues at proximity of the highly communicating E- or F-helix or C-loop.

The *communication* landscape is globally conserved in KIT mutants. Indeed, each *communication* network represents clusters formed by either highly or moderately communicating residues similarly to those in KIT^WT^ (**Figures 11** **–** **13**). The peripheral pathways formed by the lowly communicating residues are in general also cognate to those in the native KIT. Nevertheless, the *ComNets* show different communication clusters' density and boundaries in the different KIT mutants. The number of *CPs* is diminished in KIT^D816V^ (1 730), KIT^V560G^ (1 502), KIT^V560D^ (965) and KIT^D816H^ (701) respectively to KIT^WT^ (2 404), and is slightly increased in KIT^D816N^ (2 857) and KIT^D816Y^ (2 639). The number of “hubs” (44 in KIT^WT^) is diminished slightly in KIT^D816Y^ (41) and KIT^D816N^ (37), significantly in KIT^V560G^ (24) and KIT^D816V^ (25) and down to 8 and 2 in KIT^V560D^ and KIT^D816H^. These characteristics observed in KIT^D816H/V/Y^ and KIT^V560D/G^ indicate that these mutations provoke a global perturbation of communication networks.

**Figure 12:**
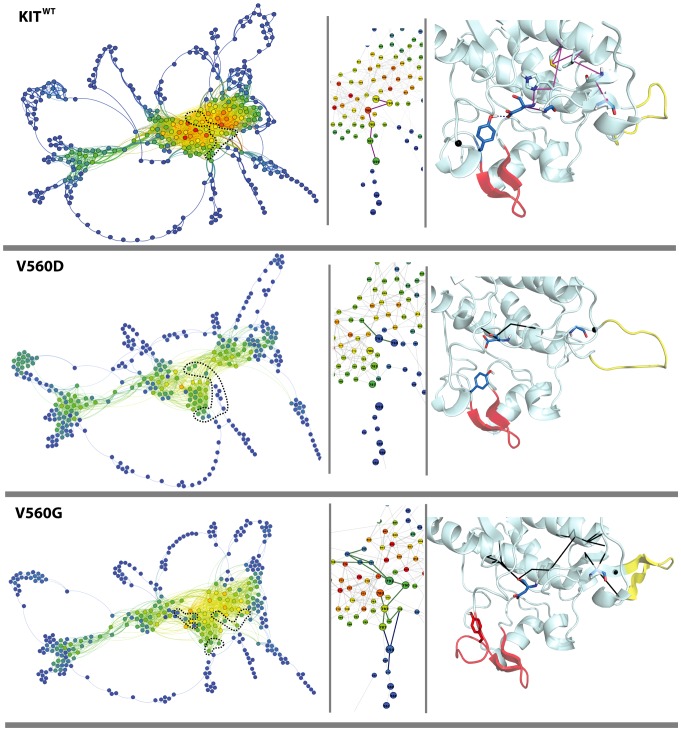
Communication pathways of cytoplasmic region in KIT^WT^ and KIT^V560G/D^ mutants. 2D and 3D graphs were drawn with Gephi and PyMOL modules incorporated in MONETA. *Left panel*: 2D graphs of a global topology illustrating the inter-residues communications in KIT^WT^ and KIT mutants. Residues are represented by points, c*ommunication pathways* (*CPs*) are depicted by bold lines and two connected residues (by at least on eCP) by a thin line. Residues are coloured from blue through green and yellow to red according to their communication efficiency. The colours are normalized according to the global range of these values for all studied proteins. The C-loop residues are contoured by dotted lines. *Middle panel*: Large-scale view of the *CPs* in KIT zoomed on *CPs* in the JMR and the A-loop. Residues are coloured from blue through green and yellow to red according to their communication efficiency. The colours are normalized according to the range of values for each protein, reflecing a relative communication efficiency of residues in each protein. The C-loop residues are contoured by dotted lines. Each residue is labelled by its number in KIT sequence. *Right panel*: Average MD conformation presented as cartoon. The residues forming *IDSs* in the A-loop and the JM-Switch are colored in red and yellow respectively. V559, D792 and Y823 are show in sticks, positions of V560G/D are indicated by black points. *CPs* initiated by either V559 or D792 are represented in bold lines, magenta in KIT^WT^ (*CPwt* only) and black in the mutants.

**Figure 13:**
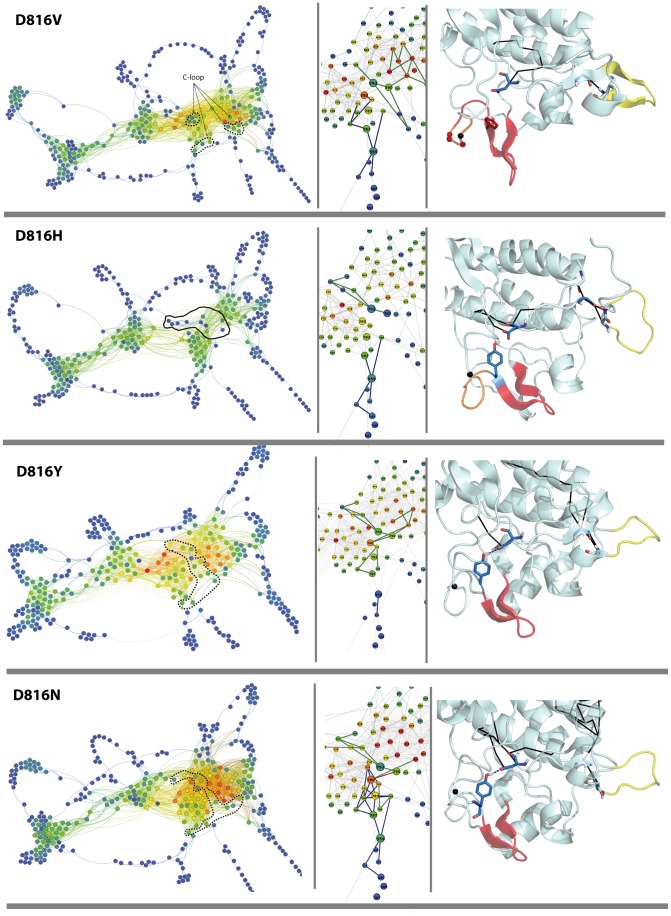
Communication pathways of cytoplasmic region in KIT^WT^ and KIT^D816N/Y/H/V^ mutants. 2D and 3D graphs were drawn with Gephi and PyMOL modules incorporated in MONETA. *Left panel*: 2D graphs of a global topology illustrating the inter-residues communications in KIT^WT^ and KIT mutants. Residues are represented by points, c*ommunication pathways* (*CPs*) are depicted by bold lines and two connected residues (by at least on eCP) by a thin line. Residues are coloured from blue through green and yellow to red according to their communication efficiency. The colours are normalized according to the global range of these values for all studied proteins. The C-loop residues are contoured by dotted lines. *Middle panel*: Large-scale view of the *CPs* in KIT zoomed on *CPs* in the JMR and the A-loop. Residues are coloured from blue through green and yellow to red according to their communication efficiency. The colours are normalized according to the range of values for each protein, reflecing a relative communication efficiency of residues in each protein. The C-loop residues are contoured by dotted lines. Each residue is labelled by its number in KIT sequence. *Right panel*: Average MD conformation presented as cartoon. The residues forming *IDSs* in the A-loop and the JM-Switch are colored in red and yellow respectively. V559, D792 and Y823 are show in sticks, positions of D816N/Y/H/V are indicated by black points. *CPs* initiated by either V559 or D792 are represented in bold lines, magenta in KIT^WT^ (*CPwt* only) and black in the mutants.

A *communication pathway* between the JMR and the A-loop in KIT^WT^ connects residue V559 (JMR) and the catalytic aspartate D792 (C-loop) through the interaction network formed by C787 (β6 in C-lobe), L783 and M780 (E-helix) and H790 (C-loop), and protrudes to Y823 (A-loop) by a highly stable H-bond D792•••Y823 (occurrences in 95% of the dynamic conformations). This extended *CP* controlled by two protein *sockets* (V559 and D792) reflects the communication between protein residues separated by 233 residues in the sequence and by more than 10 Å in the 3D structure, and involves only 6 residues which constitute a *communication fiber* between the JMR and the A-loop. This communication pathway observed in KIT^WT^ between two main regulatory fragments, the A-loop and the JMR, will be further referenced as *CP_WT_*. Residues V559 and Y823 connected by *CP_WT_* are adjacent to S2 and S8 respectively, and positioned outside the *IDSs*. Each residue, V559 or D792, participates in 9 *CPs*; the longest *CP* involves 8 residues and covers a distance of 15 Å.

Detailed analysis of KIT communication networks reveals that the *CP_WT_* is broken in all mutants at different points. Two independent non-overlapping networks, composed of pathways initiated by *sockets*, V559 (JMR) or D792 (C-loop), are systematically observed (**Figures 12** and **13**). In KIT^D816V/H^, the disruption of CP_WT_ is associated with the large extension of the *IDS* S8 in the A-loop and its partial “duplication” annotated as the *IDS* S8′ (**Figure 10**) and with H-bond between Y823 in the A-loop and D792 in the catalytic loop. The *occurrence* and *commute time* (*CT*) of D792•••Y823 are affected either partially in KIT^D816H^ (80%, 0.43) or significantly in KIT^D816V^ (40%, 1.24) respectively to KIT^WT^ (95%, 0.16). Consequently, instead of a long extended *communication pathway CP_WT_*, two separate networks are initiated by *sockets* V559 and D792 in these mutants (**Figure 13**). In KIT^D816H^ the short *CPs* formed by V559 and D792 connect only local residues of the JMR and E-helix respectively. In KIT^D816V^ the two networks are composed by *CPs* involving 5–7 residues and covering distances of 2–7 Å. In KIT^D816N/Y^ the H-bond D792•••Y823 is characterized by a very high *occurrence* (100%) and a short *commute time* (0.23/0.17), similarly to KIT^WT^. Nevertheless, the *CP_WT_* V559-Y823 is disrupted, similarly to KIT^D816V/H^. Surprisingly, despite a high global communication propensity of each *socket*, V559 and D792, there is no pathway connecting these residues. All *CPs* initiated by D792 in these mutants are either local or extended to the E-helix. The *CPs* formed by V559 in both mutants reach the F-helix.

In the JMR mutants, the H-bond D792•••Y823 shows a low occurrence and a high *commute time* (20/22% and 0.67/5.14 in KIT^V560D/G^ respectively), indicative of a partial disappearance of this non-covalent interaction. Analysis of the communication paths in KIT^V560D^ denotes only one short *CP* initiated by D792 (C-loop) (**Figures 12**). In KIT^V560G^, both *sockets*, V559 and D792, are highly connected, involved in 9 *communication pathways* of different lengths (from 2 to 8 residues). Remarkably, one *CP* from each residue V559 or D792 reaches F782 in the E-helix. This pivot residue would simultaneously participate in concerted motions (short *CTs*) with two sets of residues which themselves move independently from each other (long *CTs*). The several long *CPs* initiated by D792 are extended to either D851 or E861 in the F-helix. Similarly to KIT^D816N^, there is no path connecting the two networks controlled by communication *sockets* V559 and D792.

Overall destabilization of this communication in mutants favors an independent dynamical behavior of the JM-Switch with respect to the kinase domain, producing an unconstrained structural arrangement of the JMR, as we evidenced by structural analysis, conformational sampling and PCA. Remarkably, the structure and the dynamical behavior of the A-loop seems quantitatively more affected by mutation D816V than D816H, as was evidenced by RMSFs profiles, PCA, structural analysis and the *IDSs*. The level of these effects is in excellent correlation with the degree of the H-bond D792•••Y823 decrease and with a more apparent structural modification of the JM-Switch induced by mutation D816V than by mutation D816H. It is also worth noting that in the mutants KIT^D816Y/N^, which display very similar *ComNets* profiles, as well as nearly similar structural and dynamical features, the local (and partial) destabilization of the A-loop structure does not influence the A-loop *IDS*. However, the tiny perturbation of the A-loop structure seems “communicated” all the way to the C-loop through the conserved H-bond D792•••Y823 and negatively affects the communication between the C-loop, the E- and F-helices and the residue V559. Due to this perturbed communication the structural and dynamical features of the JM-Switch are affected similarly in KIT^D816N/Y^, however in a smaller yield.

For the JMR mutants, our data, indicating a good correlation between the structural and dynamical manifestations induced by mutation V560G/D, reveal the perturbation of the communication initiated by the JMR *socket* V559, the residue adjacent to the mutation site, and the disruption of the D792•••Y823 H-bond. The stability of this H-bond interaction between the residues from the A- and C-loop seems negatively correlated with either the long-range or short-range effects of the mutation on the JM-Switch folding and dynamics. The auto-inhibitory position of the JM-Switch in KIT^WT^ depends on a finely regulated *communication pathway* involving the JM-Switch, the E- and F-helix and the C- and A-loop. A mutation that favors an independent behavior of one of the segments involved in the *communication pathway CP_WT_* provokes a perturbation of the dynamical equilibrium of this *CP* which in turn disturbs the auto-inhibition of the kinase domain by the JMR.

## Discussion

The allosteric nature of receptor tyrosine kinases regulation was established more than a decade ago from functional studies [81], [82]. This regulation includes a sequence of multistep responses of the receptor to a perturbation. Binding of a specific ligand (SCF for KIT) to the extracellular domain induces large conformational changes, leading to extracellular dimerization. The perturbation propagates through the membrane via a single transmembrane helix, inducing activation of the cytoplasmic kinase domains. The activated kinase domains promote transphosphorylation of specific tyrosine residues, initiating cascading events. Phosphorylation leads to major conformational rearrangements, forming phosphotyrosine binding (or docking) sites in the JMR and kinase insert domain (KID) for recruitment of downstream SH2 domains and phosphotyrosine or serine-binding (PTB) domains. Such a ligand-controlled activation can be described in terms of a system response to orchestrated perturbations, the ATP- or phosphate binding, in varied spatio-temporal scales. A tightly regulated linkage between the functional sites of receptor (sites coupling) demonstrates the allosteric character of the activation mechanisms.

The mechanisms of allosteric regulation in non-receptors protein kinases were explored by computational studies. They are largely documented for c-Src [83]–[89] [90]–[92] and limited to seldom reported works for ABL [93], CDK5 [94], PKA [95], AKT/PKB [96], RET and MET [97] and adenylate kinase [98]. Regulation of receptor tyrosine kinases activation, principally for the epidermal growth factor receptor (EGFR) [51], [99]–[104] and the stem cell factor receptor KIT [105] was also characterized at the atomic level. All these *in silico* studies have suggested that functional coupling between collective motions and local structural changes can explain the experimental data and provide molecular insights into allosteric mechanisms in the tyrosine kinases. We have previously contributed to the understanding of the allosteric mechanism in the native KIT by detection and characterization of a *communication pathway* between the A-loop and the JMR [41].

Moreover, we have reported that the impact of the mutation D816V on conformational dynamics of KIT spreads far beyond the mutation site leading to functionally significant changes in structure and conformational mobility at the remote JMR [40]. The extensive *in silico* characterization of the structural and dynamical effects induced by gain-of-function mutations localized either in the A-loop or in the JMR of KIT, two key elements engaged in regulation of the central receptor functions – kinase activity and intracellular signalling – considerably completed our preliminary study. We evidenced that the polymorphic substitution of a negatively charged residue (D) in position 816 to an alkaline (D816H), polar (D816N), aromatic (D816Y) or aliphatic (D816V) residue influences strongly the secondary and tertiary structure of KIT. The mutation-induced effects evidenced as structural reorganization in the A-loop, the protein fragment where the mutation is localized (local or short-range effect), and in the JMR, a protein fragment distant by more than 15 Å from the point mutation (long-range effect), are manifested in the all studied A-loop mutants. The magnitude of these effects is not equivalent in the analyzed mutants and depends on the type of substitution at the mutation site.

Do the observed effects in KIT are induced by the mutation? Could we use MD simulations as a valid and sensitive approach to distinguish fine effects in the mutants? To examine this, we carried out a test for reproducibility of allosteric control in the reverse mutant. Our study of the reverse mutation effects (mutant back to normal) in KIT D816H showed that the structural features of a KIT (WT) model derived from the mutant are very close to those observed in the native protein [106] (**Figure S5**). In the reverse mutant the A-loop and JMR folding is regulated by the restored allosteric communication between these distant regions. This finding represents, to our knowledge, the first instance of a structural evidence of reversibility of mutation-induced consequences at the molecular level.

KIT hotspot mutations in the intracellular JMR, involved in maintaining the inactive state of receptor tyrosine kinases (the auto-inhibitory function of JMR), induced only local effects on the JM-Switch. In both JMR-mutants, the JM-Switch is displaced from its packed-to-the-C-lobe conformation observed in KIT^WT^ towards an axial position in respect to the kinase domain, similarly to the A-loop mutants. This conformational rearrangement goes along with a spectacular change in the JM-Switch β-folding, either largely stabilized (V560G) or completely abolished (V560D). Therefore, we consider a disturbing effect on the JM-Switch folding as indicative of the JMR departure from its inhibitory position and general effect induced by KIT hotspot mutations. Such departure of JMR from the kinase domain favors the stabilization of the inactive non-autoinhibited state [107], [108], where the JMR displays an increased solvent accessibility, as was revealed by hydrogen/deuterium exchange mass spectrometry analysis of KIT^D816H^ and KIT^V560D^ mutants [107]. Nevertheless, the contribution of this change to KIT activation is different in the two kinds of mutation – in the A-loop and in the JMR. The different order of magnitude of the mutation-induced structural effects and the distinct intramolecular communication networks put in evidence a strong sensitivity of KIT regulation to (i) the positions of gain-of-function mutations, the A-loop and the JMR, and to (ii) the nature of residue at the mutation site. The mutation-induced activation seems is regulated by peculiar allosteric mechanisms and can produce distinct dysfunctions of KIT.

As the structural effects induced by the activating mutations in KIT were mainly detected in fragments crucial for the activation/deactivation mechanisms, they should be related to the activation rates and clinical occurrences of these mutants. The most pronounced structural effects in the A-loop are induced by the D816V mutation, which is the most clinically observed primary mutation in patients with mastocytosis [109] and GISTs [29] and is also detected at lower frequencies in acute myeloid leukemia and in germ cell tumors [110]. This mutation results in ligand-independent constitutive kinase activity characterized by a considerably increased auto-activation rate (by a factor 536) respectively to the native receptor, whereas the D816H mutation induces a more moderate increase (by a factor 184) in absence of SCF [30]. Nevertheless, the KIT^D816H^ auto-activation rate is significantly higher than that of KIT^D816N^ [25]. The KIT^D816Y/N^ mutants showing moderate mutation-induced effects are only rarely detected in clinic as primary pathogenic mutations. V560G/D mutations represent the majority of KIT mutations found in GISTs [14] and melanoma [111]. The impact of V560D/G mutation on JMR is also more pronounced than D816V/H mutation, consistent with a higher activating potency of this substitution [15], [30]. Such relationships of the structural effects and the *in vitro* (and/or *in vivo*) activation rates (and/or pathogenicity) of these mutants confirm the activation mechanism that we proposed earlier for KIT^D816V^.

**Figure 14:**
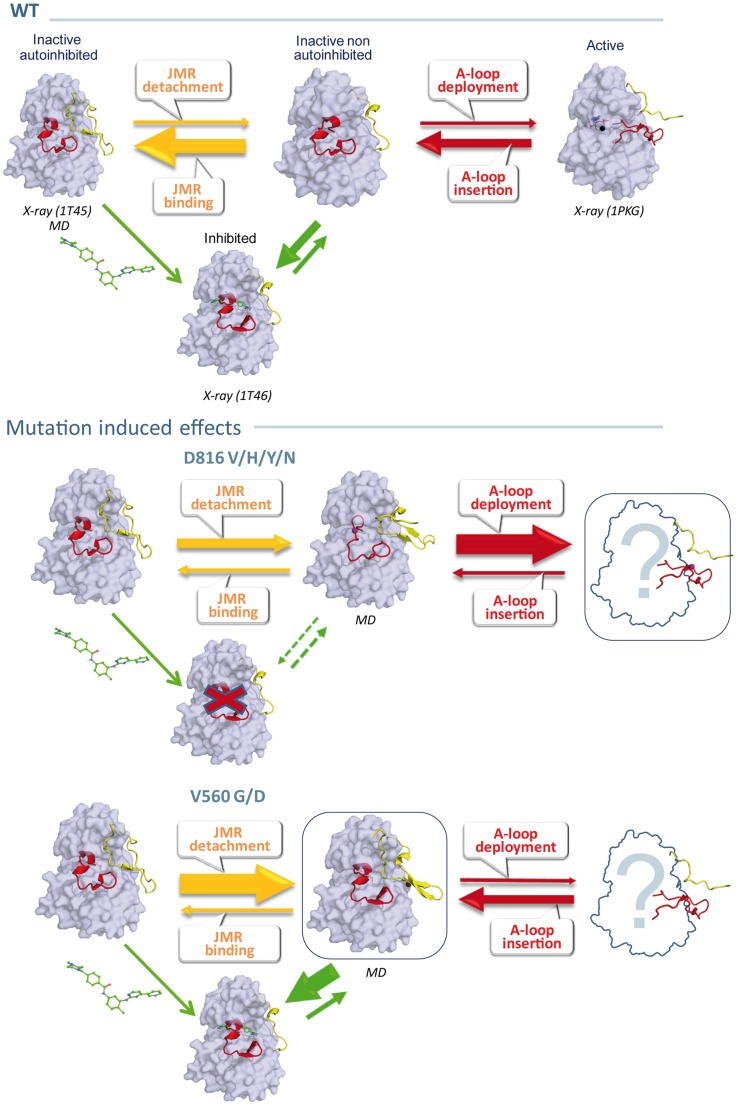
Proposed mechanisms of the constitutive activation of KIT mutants and consequences on drug sensitivity. The multi-states equilibrium of KIT cytoplasmic region in KIT^WT^ (*upper panel*), KIT^D816H/V/Y/N^ (*middle panel*) and KIT^V560G/D^ (*lower panel*). Each KIT conformation is represented as a molecular surface, except the JMR and the A-loop and imatinib drawn as cartoons and sticks respectively. In KIT mutants, the mutation position is shown by a ball. Equilibrium between two states is denoted by arrows of different thicknesses. *Upper panel*: In the absence of SCF, KIT^WT^ is mainly in the inactive autoinhibited state maintained by the JMR non-covalently bounded to the kinase domain. This state of KIT is the imatinib target. *Middle panel*: The A-loop mutations (D816V/H/Y/N) induce the inactive non-autoinhibited state of KIT evidenced by the JMR departure from the kinase domain. This effect conducts to deployment of the A-loop eventually leading to the constitutively active KIT state. The inactive non-autoinhibited state is not a suitable target for imatinib that inhibit the inactive autoinhibited state. *Lower panel*: The JMR mutations (V560G/D) greatly impact the JMR binding to the kinase domain and facilitate its departure, favoring the non-autoinhibited state, whereas the inactive conformation of the A-loop is still conserved. The inactive non-autoinhibited state of KIT is more consented in KIT^V560G/D^ than in KIT^WT^ and especially in KIT^D816V/H/Y/N^, leaded to the increased sensitivity of KIT^V560G/D^ to inhibitor compared to KIT^WT^. In each panel, the most preferred state of KIT in the presence of imatinib is encircled.

Reported biochemical data indicate strong changes in the sensitivity of KIT mutants to inhibitors type II (*e.g*., imatinib) compared to KIT^WT^. Particularly, the A-loop mutation D816V/H/Y/N induces KIT resistance [28], [38]. The structural response of KIT to the perturbation caused by the hotspot mutation D816H/V/N/Y stimulated our study of the effects induced by resistance-inducing but not activating mutation D802V in another tyrosine kinase receptor of KIT sub-family, CSF1-R. Mutation D802V in CSF1-R is equivalent to D816V in KIT and was reported as stabilizing the active kinase domain structure to which imatinib does not bind [34], [112], as suggested by the resulting resistance to imatinib. As the equivalent mutations (D816V and D802V) are both associated to resistance outcome *in vitro*, a similar *in silico* behavior would be expected from both receptors. A similarly conducted *in silico* study showed that the local impact of D802V mutation manifested as a partial unfolding of small 3_10_-helix at proximity of point mutation in CSF-1R is equivalent to that observed in KIT mutant [113]. However the long-range mutation effect evidenced in KIT was not observed in CSF1-R. This observation proves once again that destabilisation of the A-loop conformation is a major factor contributing to loss of sensibility to the drugs. In the A-loop mutants of KIT, the partial destabilization of the A-loop conformation evidenced by our *in silico* study may contribute to the complete unfolding of the A-loop, following the departure of JMR from the active site non-observed in CSF1-R. Thereby this mutation would facilitate KIT transition toward a fully active conformation by altering the conformational equilibrium of the kinase toward the active form which compromises the efficacy of the inhibitors targeting inactive KIT (**Figure 14**).

In contrast, the JMR mutations V560D/G increase the drug sensitivity of the receptor compared to the wild type receptor [15], [30]. It was shown that imatinib binds to the inactive non-autoinhibited state in which the JMR is detached from the kinase domain [107], [108]. The inactive non-autoinhibited conformations of KIT^V560G/D^ mutants, which is induced by JMR detachment from the kinase domain, would be the most appropriate targets for imatinib binding. The common effect of both mutation types (V560G/D or D816V/H/Y/N) on the JMR behavior but different effect on A-loop conformational stability, may explain the high efficiency of imatinib inhibition on the JMR-mutants but low efficiency on the A-loop-mutants.

As these disease-related mutations are far away from the binding sites (SCF- or ATP-binding sites) of KIT, their action can be governed by allosteric mechanisms and may perturb signal propagation. In this context a mutation can be considered as a transforming perturbation of the functional intra-molecular network. Correlation between a local effect evidenced as the destabilization of the small A-loop 3_10_-helix, and long-range structural effects proves the allosteric nature of intermolecular regulation between A-loop and JMR in KIT mutants. Identification of the communication pathways in protein using a dual formalism – topological descriptors and dynamical correlations – provides physically significant elements to depict the signal (or perturbation) propagation between residues [41].

We used the *Independent Dynamics Segments*, introduced by R. Nussinov [79] and later referred as *Discrete Breathers* [114] to define protein segments with distinct dynamical properties. These fragments are very important for protein functions, particularly for the energy relocation over conformational transition and/or in allosteric propagation [115]–[117]. *IDSs* were found in the main regulatory fragments of the cytoplasmic domain of KIT – in the A-loop, the C-helix and N-C-loop, the JM-Switch and the N-terminal part of the JMR which were reported to undergo conformational change upon KIT activation [3]. Particularly, the C-terminal part of the JMR constitutes the hinge for JMR pivoting upon activation, the G-helix and N-G-loop constitute a storage platform for substrates in KIT active state and adapts their conformation to the stored substrate [108]. The short and solvent accessible flexible loops linking rigid β-stranded segments are also contain the *IDSs* displaying independent motions. Alteration of the *IDSs* profile in KIT mutants indicates to an important transformation of the regulatory fragments functions, and a global disappearance of locally coupled dynamical behaviors in the mutants.

We further identified chains of non-covalently linked and dynamically coupled residues constituting *communication fibers* across the protein network between *IDSs*. The crucial communication pathway between the A-loop and the JMR through the catalytic loop (C-loop) was interrupted in mutants and replaced by other communication pathways successfully regulating the constitutive activity of KIT. All studied KIT gain-of-function mutations prompt novel paths controlling KIT activation through the residues highly connected to the others, the “hubs”, positioned on the functionally meaningful fragments, mainly the E and F-helix.

The interruption of communication between the A-loop and the JMR allows a self-governed unconstrained JM-Switch fold in KIT mutants, ordered mainly by the local properties of the JMR polypeptide sequence. Consequently, the randomly coiled structure of the JM-Switch in KIT^WT^ is regulated by the allosteric communication within the cytoplasmic domain, established particularly between the A-loop and the JMR. The subtly folded and rather flexible JMR controls a facile entry of the JM-Binder fragment in the active site of the native protein, maintaining the A-loop in the inactive conformation. The natural SCF-induced activation transmitted by the allosteric communication of the extracellular domain and the kinase domain and prompting the JMR departure from its autoinhibitory position in KIT^WT^, is replaced in KIT mutants by a mutation-induced activation evidenced by promotion of the inactive non-autoinhibited state of the cytoplasmic domain, which in turn would favor a conformational transition toward the fully active conformation. In contrast to the reversible activation/deactivation process initiated by ligand SCF in the native KIT, the mutation-induced activation is quite irreversible. The gain-of-function mutation acts as a perpetual effector induced a particular state leading to KIT constitutive activation.

### Conclusions

We described the effects induced by the gain-of-function mutations on structure, dynamics and intra-protein communication of KIT as a function of the mutation location and of the residue nature in the mutation site. Our study is based on the exploration of the conformational space of the studied proteins limited by the modest duration of our MD simulations. Nevertheless we observed the effects involving local and medium-scale motions of the functionally crucial protein fragments. The JMR departure from its auto-inhibitory position – a commonly observed mutation-induced effect in KIT – constitutes the first triggering step of protein kinase domain activation. We proved that all studied hotspot mutations stabilize an inactive non-autoinhibited state of KIT, promoting receptor conformational shift towards an active conformation and leading to the KIT constitutive activation. The obtained results allow us to postulate activation mechanisms in KIT^D816H/Y/N/V^ and KIT^V560G/D^, depicted on **Figure 13**. The destabilization of the A-loop structure in KIT^D816H/Y/N/V^, evidenced by increased of its partial unfolding, plausibly corresponds to an earlier conformational change of the A-loop over the activation process. The newly observed A-loop conformations, identified in MD simulations of KIT^D816H/Y/N/V^ mutants, differ from KIT^WT^ conformations and depict an intermediate state between inactive and active states. Similarly to the long-range effects induced by the A-loop mutations, the JMR mutations also promote conformational transition of KIT towards a non-autoinhibited state characterized by the JMR detached from kinase domain. Our *in silico* analysis established the correlations between the structural and dynamical effects induced by V560G/D (JMR) or D816H/Y/N/V (A-loop) mutations and the auto-activation rates of mutants measured *in vitro* and *in vivo*.

Further, the proper effect of the A-loop and the JMR mutations consists of drug sensitivity alteration. The structure of the A-loop mutants depicts an intermediate state between inactive and active KIT conformations that may prevent the binding of inhibitors targeting the inactive non-autoinhibited state and consequently represents the drug resistant form. The mutation effect on JMR observed in KIT^V560G/D^ mutants is not associated with the local conformational destabilization of the A-loop, and may facilitate inhibitors binding to the active site thus increasing sensitivity of KIT to kinase inhibitors. Indeed, the JMR mutants are known to be more sensitive to many kinase inhibitors than KIT^WT^, whereas the A-loop mutants are essentially resistant [15], [30].

The established cross-correlations between the local and long-range structural and dynamical effects in KIT, prove the allosteric character of the gain-of-function mutations action. Description of the structural effects and of the physical support for allosteric coupling/decoupling provides the basic data for the mechanisms of KIT constitutive activation and contributes to the understanding of allosteric regulation in this protein. The described conformations of KIT represent novel targets for drug design.

## Supporting Information

Figure S1 **MD simulations of** KIT^D816N^ **in the inactive state.** The RMSDs (in Å) per residue were calculated from trajectories **1** (red) and **2** (green) of MD simulations on backbone residues 551–928.
(TIF)

Figure S2 **MD simulations of KIT cytoplasmic domain in the inactive state.** The RMSDs (in Å) per residue were calculated from trajectories 1 (red) and 2 (green) of MD simulations of KIT^WT^, KIT^D816V^, KIT^D816H^, KIT^D816Y^, KIT^D816N^, KIT^V560G^ and KIT^V560D^ on the backbone atoms of A-loop (left) and JMR (right).
(TIF)

Figure S3 **RMSFs in KIT^D816Y^, KIT^D816N^, KIT^D816H^, KIT^D816V^, KIT^V560G^ and KIT^V560G^.** The proteins are presented as tubes: the KIT regions or fragments are displayed with different colors - JMR (yellow), A-loop (red), N- and C-lobe (cyan and blue) and KID (gray). The size of tube is proportional to the difference in the by-residue atomic fluctuations in KIT^WT^ and KIT mutants computed on the backbone atoms.
(TIF)

Figure S4 **Amplitude of collective motions of the JM-Switch backbone, in the first 5 modes of each simulated model.** (**a**, **b**) Each model correspond to one histogram bar, in which each portion (from dark to pale grey) corresponds to one mode (from 1^st^ to 5^th^) and represents by its height the norm of the resultant of the JM-Switch backbone motion weighted by the contribution of the mode to the global motion (eigenvalue). On histogram (b), these values are also weighted by the contribution to the mode of the non-*pseudo*-KID residues. (**c**) Contribution of the motions of *pseudo*-KID residues to the global motion of KIT in each of the 5 first modes for each model.
(TIF)

Figure S5 **MD study of KIT cytoplasmic region in the native KIT (KIT^WT^), its D816H mutant (KIT^D816H^) and the reverse H816D mutant (KIT^H816D^).** (**a**) Superposed conformations of KIT^WT^ and KIT^H816D^ were selected by RMSDs clustering (cutoff of 2.5 Å). Ribbon diagrams display the proteins regions or fragments with different colors: JMR (yellow in KIT^WT^ and orange in KIT^H816D^), A-loop (red in KIT^WT^ and salmon in KIT^H816D^), N-lobe (cyan in KIT^WT^ and pale cyan in KIT^H816D^), C-helix in the N-lobe (dark green in KIT^WT^ and green in KIT^H816D^), C-lobe (marine in KIT^WT^ and light blue in KIT^H816D^), G-helix in C-lobe (magenta in KIT^WT^ and pink in KIT^H816D^), KID (grey in KIT^WT^ and lightgray in KIT^H816D^). (**b**) Secondary structures in the cytoplasmic region of KIT^WT^, KIT^D816H^ and KIT^H816D^. Secondary structure assignments for the JMR (left) and the A-loop (right) were averaged over the two replica of each MD simulations (2×48 ns) of KIT^WT^ and mutants. For each residue, the proportion of each secondary structure type is given as a percentage of the total simulation time and shown with lines of different colors: 3_10_-helices (red), antiparallel β-sheet (blue), turns (green), total structure (dashed gray).
(TIF)

Table S1 **Preparation details of the MD simulations.** Data for KIT^WT^ and KIT^D816V^ reported previously [40] are distinguished in grey. Root mean square deviations (RMSDs) of each model from the initial template were computed on backbone atoms. The counter-ions Na^+^ was employed to neutralize the systems.
(DOC)

Table S2 **RMSDs values (mean, standard deviation and maximum, in Å) computed on the backbone atoms of KIT cytoplasmic region in the inactive form.** The RMSDs were calculated from two independent MD simulations for each mutant, with respect to the initial frame. Data for KIT^WT^ and KIT^D816V^ reported previously [40] are distinguished in grey.
(DOCX)

Table S3 **Convergence analysis data on the two MD trajectories of wild-type and mutated KIT.** Data for KIT^WT^ and KIT^D816V^ reported previously [40] are distinguished in grey. The convergence criterion (*c*) was calculated as described in ***Materials and Methods***.
(DOC)

Table S4 **The A-loop and the JM-Switch internal H-bond network in KIT^WT^ and KIT^D816V/H/Y/N^.** Only the H-bonds which time occupancy differs significantly among the simulated models are presented. The residue and its fragment, the side chains – sc – or the backbone – bb, involved in the A-loop and JMR internal H-bonding are shown.
(DOC)

Table S5 **Distance and angle measurements.** The distance D_JM**S**_ between the JM-Switch and the center of mass of residues 847 and 912 in the C-lobe, the angle A_JMS_ drawn by the JM-Switch, the PKD and the C-lobe, and the distance D_A_ between the A-loop and the rest of PKD where measured every 10 ps and averaged upon 2×48-ns or 2×65-ns of productive simulation time.
(DOC)
