# Supplementary figures and images for: Hotspot Mutations in KIT Receptor Differentially Modulate Its Allosterically Coupled Conformational Dynamics: Impact on Activation and Drug Sensitivity
Source: PLoS Comput Biol. 2014 Jul 31;10(7):e1003749. doi: 10.1371/journal.pcbi.1003749 (PMC4117417; doi:10.1371/journal.pcbi.1003749)

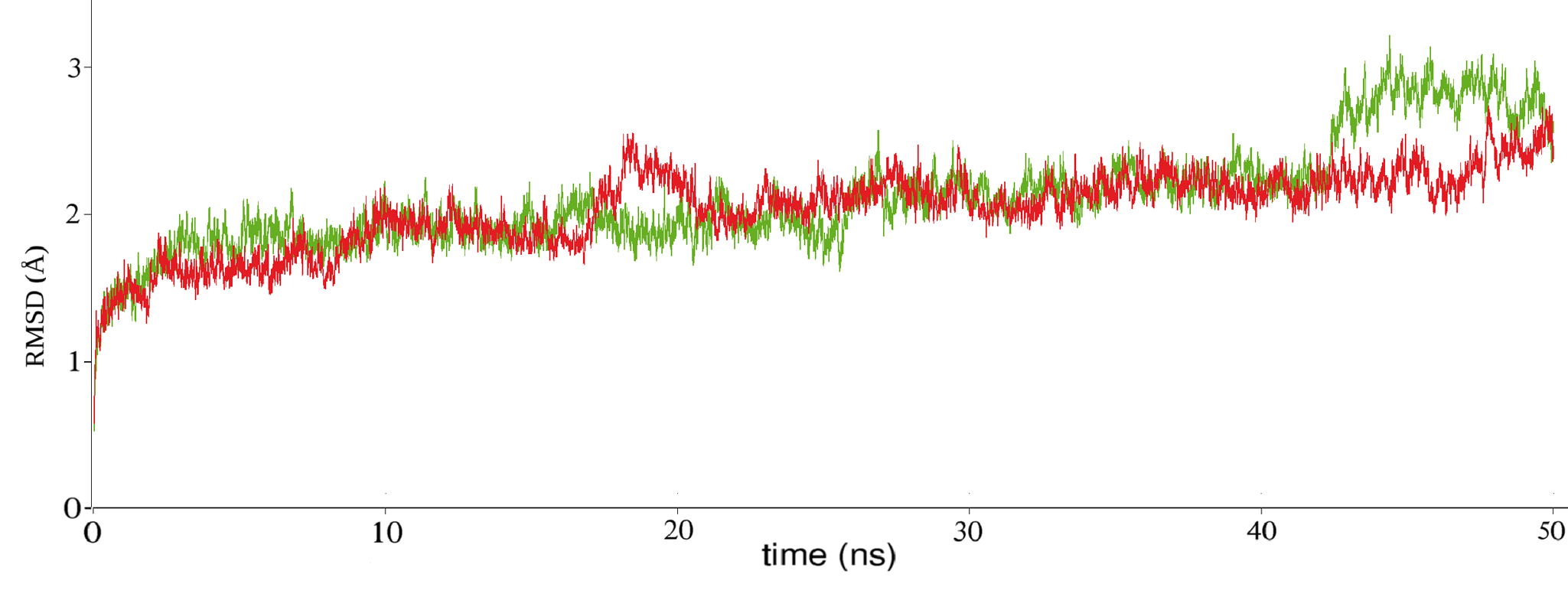

Supplement: Figure S1 — MD simulations of KITD816N in the inactive state. The RMSDs (in Å) per residue were calculated from trajectories 1 (red) and 2 (green) of MD simulations on backbone residues 551–928. (TIF) [file pcbi.1003749.s001.tif]

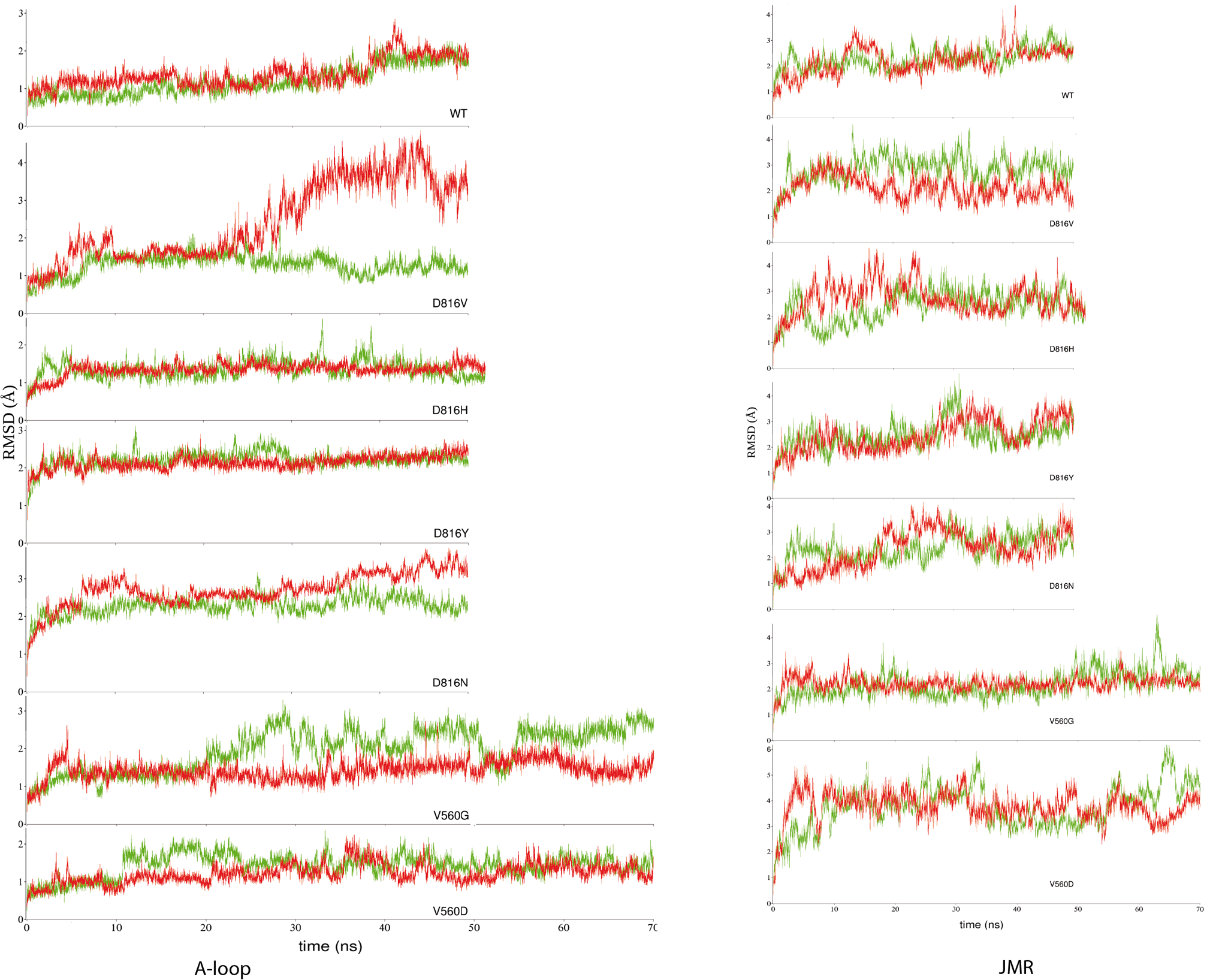

Supplement: Figure S2 — MD simulations of KIT cytoplasmic domain in the inactive state. The RMSDs (in Å) per residue were calculated from trajectories 1 (red) and 2 (green) of MD simulations of KITWT, KITD816V, KITD816H, KITD816Y, KITD816N, KITV560G and KITV560D on the backbone atoms of A-loop (left) and JMR (right). (TIF) [file pcbi.1003749.s002.tif]

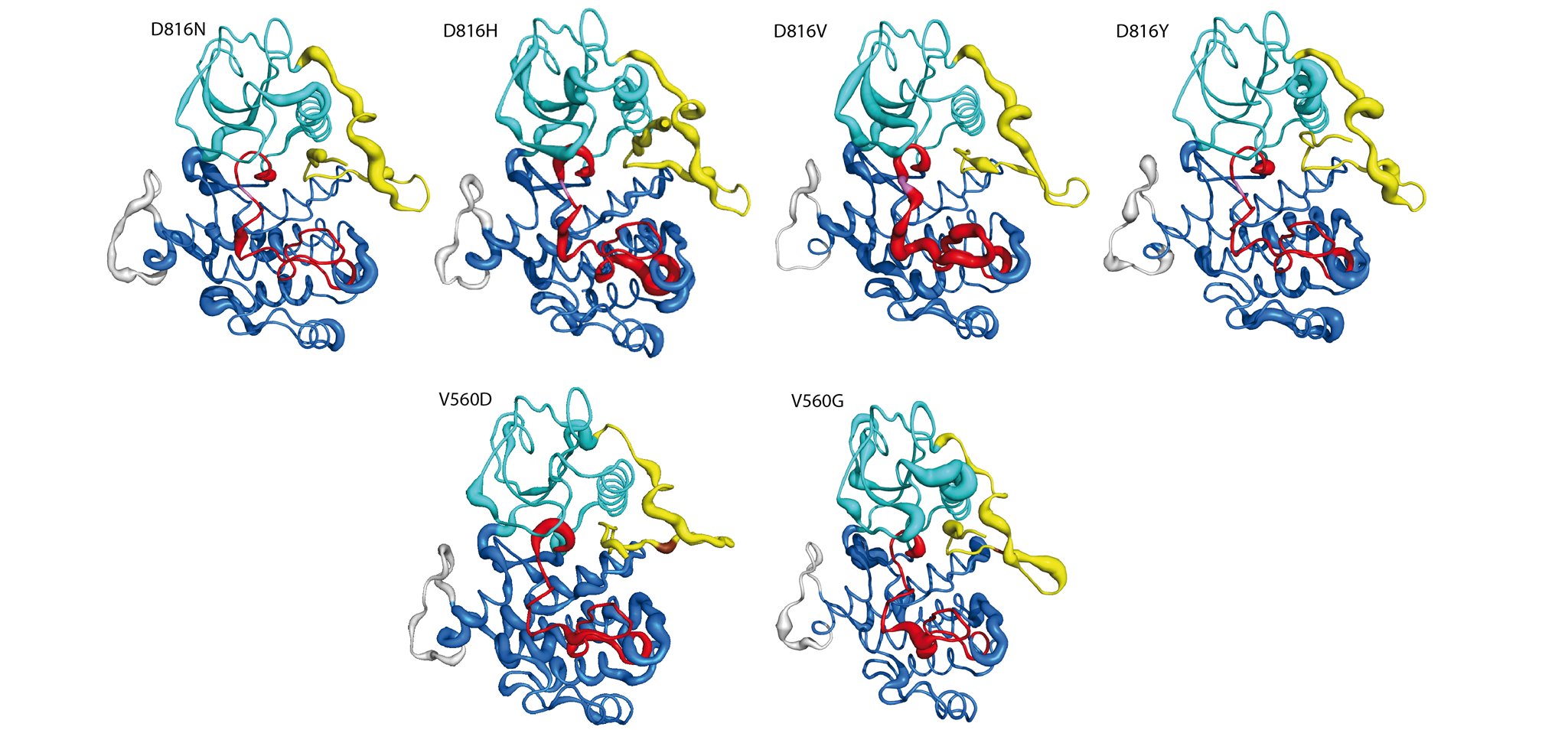

Supplement: Figure S3 — RMSFs in KITD816Y, KITD816N, KITD816H, KITD816V, KITV560G and KITV560G. The proteins are presented as tubes: the KIT regions or fragments are displayed with different colors - JMR (yellow), A-loop (red), N- and C-lobe (cyan and blue) and KID (gray). The size of tube is proportional to the difference in the by-residue atomic fluctuations in KITWT and KIT mutants computed on the backbone atoms. (TIF) [file pcbi.1003749.s003.tif]

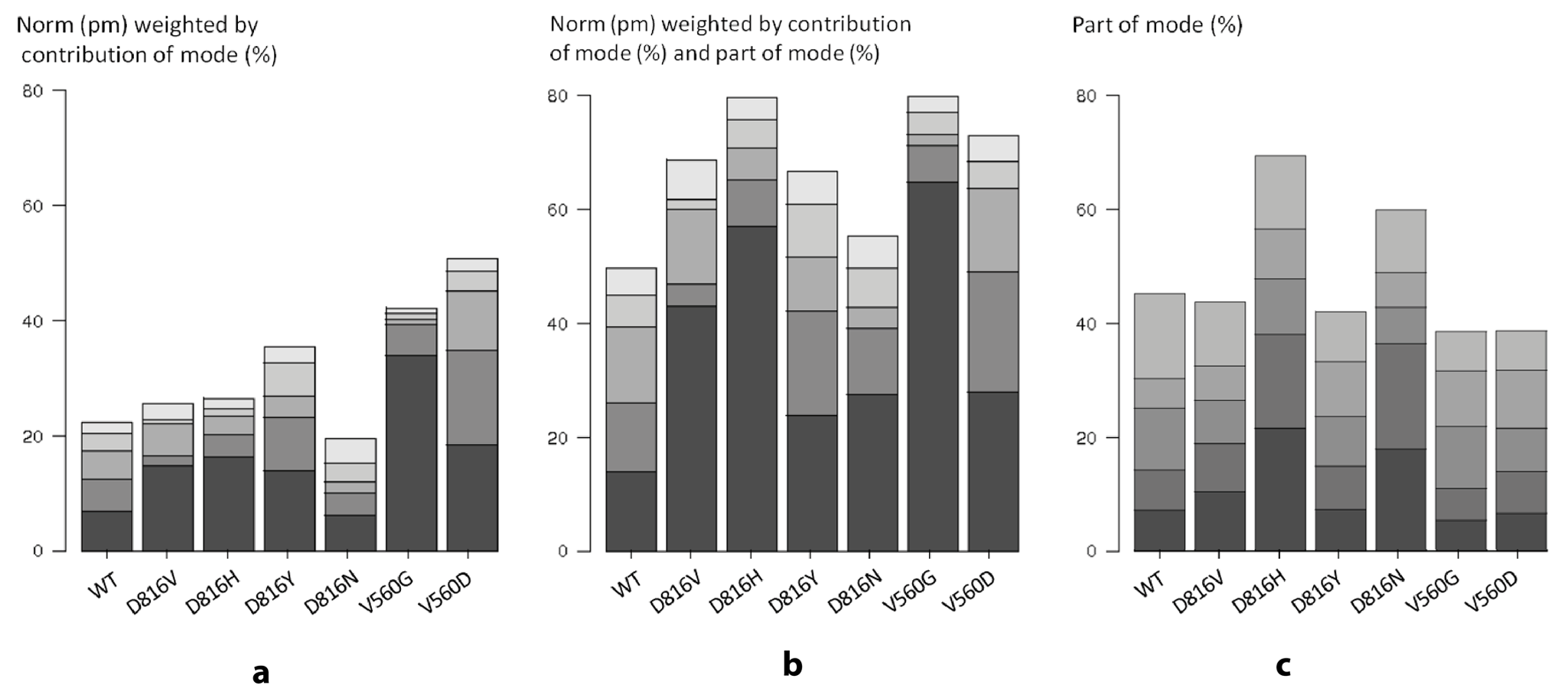

Supplement: Figure S4 — Amplitude of collective motions of the JM-Switch backbone, in the first 5 modes of each simulated model. (a, b) Each model correspond to one histogram bar, in which each portion (from dark to pale grey) corresponds to one mode (from 1st to 5th) and represents by its height the norm of the resultant of the JM-Switch backbone motion weighted by the contribution of the mode to the global motion (eigenvalue). On histogram (b), these values are also weighted by the contribution to the mode of the non-pseudo-KID residues. (c) Contribution of the motions of pseudo-KID residues to the global motion of KIT in each of the 5 first modes for each model. (TIF) [file pcbi.1003749.s004.tif]

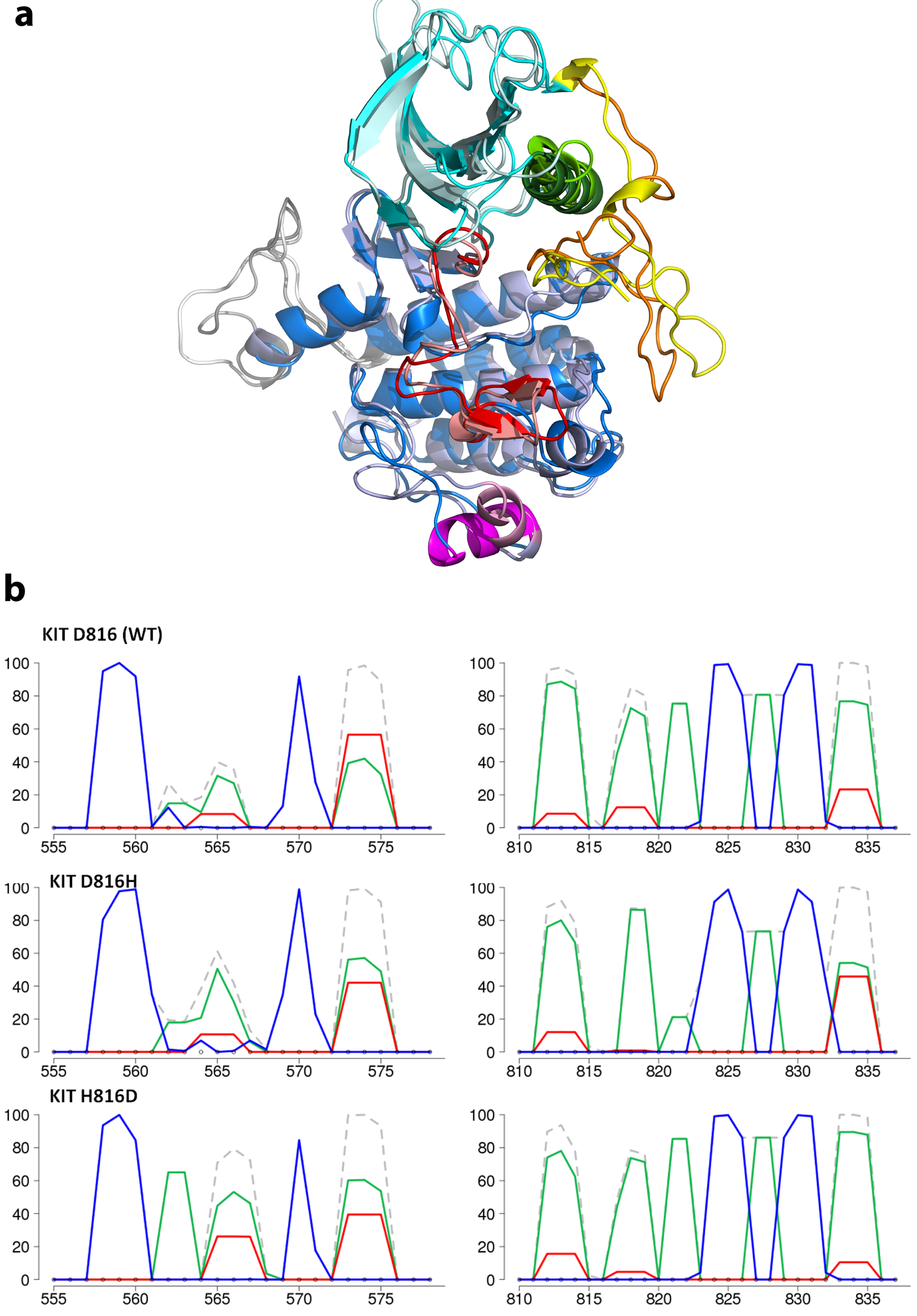

Supplement: Figure S5 — MD study of KIT cytoplasmic region in the native KIT (KITWT), its D816H mutant (KITD816H) and the reverse H816D mutant (KITH816D). (a) Superposed conformations of KITWT and KITH816D were selected by RMSDs clustering (cutoff of 2.5 Å). Ribbon diagrams display the proteins regions or fragments with different colors: JMR (yellow in KITWT and orange in KITH816D), A-loop (red in KITWT and salmon in KITH816D), N-lobe (cyan in KITWT and pale cyan in KITH816D), C-helix in the N-lobe (dark green in KITWT and green in KITH816D), C-lobe (marine in KITWT and light blue in KITH816D), G-helix in C-lobe (magenta in KITWT and pink in KITH816D), KID (grey in KITWT and lightgray in KITH816D). (b) Secondary structures in the cytoplasmic region of KITWT, KITD816H and KITH816D. Secondary structure assignments for the JMR (left) and the A-loop (right) were averaged over the two replica of each MD simulations (2×48 ns) of KITWT and mutants. For each residue, the proportion of each secondary structure type is given as a percentage of the total simulation time and shown with lines of different colors: 310-helices (red), antiparallel β-sheet (blue), turns (green), total structure (dashed gray). (TIF) [file pcbi.1003749.s005.tif]
