# Supplementary material for: Hotspot Mutations in KIT Receptor Differentially Modulate Its Allosterically Coupled Conformational Dynamics: Impact on Activation and Drug Sensitivity
Source: PLoS Comput Biol. 2014 Jul 31;10(7):e1003749. doi: 10.1371/journal.pcbi.1003749 (PMC4117417; doi:10.1371/journal.pcbi.1003749)
Supplement: Table S1 — Preparation details of the MD simulations. Data for KITWT and KITD816V reported previously [40] are distinguished in grey. Root mean square deviations (RMSDs) of each model from the initial template were computed on backbone atoms. The counter-ions Na+ was employed to neutralize the systems. (DOC) [file pcbi.1003749.s006.doc]

|  | **WT** | **D816V** | **D816H** | **D816Y** | **D816N** | **V560G** | **V560D** |
| --- | --- | --- | --- | --- | --- | --- | --- |
| Counterions | 1 Na^+^ | Ø | Ø | Ø | Ø | 1 Na^+^ | 2 Na^+^ |
| Number of protein atoms | 5284 | 5288 | 5289 | 5293 | 5286 | 5275 | 5280 |
| Water box dimensions (Å^3^) | 77.7×73.8  ×81.9 | 76.7×72.8  ×80.9 | 91.3×81.7  ×87.1 | 78.1×74.2  ×82.2 | 78.1×74.1  ×82.3 | 88.3×83.5  ×92.5 | 88.3×84.4  ×92.5 |
| Number of water molecules | 13 195 | 13 197 | 15 956 | 13 194 | 13 198 | 16 928 | 17 180 |
| Total number of atoms | 44 870 | 44 879 | 53 157 | 44 875 | 44 880 | 56 059 | 56 820 |
| Deviation after equilibration (Å) | 1.29 | 1.13 | 1.25 | 1.20 | 0.62 | 1.08 | 1.17 |
