# Supplementary material for: Hotspot Mutations in KIT Receptor Differentially Modulate Its Allosterically Coupled Conformational Dynamics: Impact on Activation and Drug Sensitivity
Source: PLoS Comput Biol. 2014 Jul 31;10(7):e1003749. doi: 10.1371/journal.pcbi.1003749 (PMC4117417; doi:10.1371/journal.pcbi.1003749)
Supplement: Table S2 — RMSDs values (mean, standard deviation and maximum, in Å) computed on the backbone atoms of KIT cytoplasmic region in the inactive form. The RMSDs were calculated from two independent MD simulations for each mutant, with respect to the initial frame. Data for KITWT and KITD816V reported previously [40] are distinguished in grey. (DOCX) [file pcbi.1003749.s007.docx]

| **Model** | **mean (Å)** | **std (Å)** | **max (Å)** |
| --- | --- | --- | --- |
| **WT** | 2.77 / 2.51 | 0.52 / 0.54 | 3.80 / 4.04 |
| **D816V** | 2.51 / 2.35 | 0.33 / 0.34 | 3.24 / 3.37 |
| **D816H** | 2.89 / 2.82 | 0.58 / 0.45 | 4.14 / 4.15 |
| **D816Y** | 2.16 / 2.26 | 0.26 / 0.35 | 2.94 / 3.30 |
| **D816N** | 2.67 / 2.32 | 0.79 / 0.40 | 5. 10 / 3.34 |
| **V560G** | 3.22 / 2.89 | 0.48 / 0.47 | 4.18 / 3.88 |
| **V560D** | 2.81 / 2.57 | 0.47 / 0.36 | 3.93 / 3.73 |
