# Supplementary material for: Hotspot Mutations in KIT Receptor Differentially Modulate Its Allosterically Coupled Conformational Dynamics: Impact on Activation and Drug Sensitivity
Source: PLoS Comput Biol. 2014 Jul 31;10(7):e1003749. doi: 10.1371/journal.pcbi.1003749 (PMC4117417; doi:10.1371/journal.pcbi.1003749)
Supplement: Table S3 — Convergence analysis data on the two MD trajectories of wild-type and mutated KIT. Data for KITWT and KITD816V reported previously [40] are distinguished in grey. The convergence criterion (c) was calculated as described in Materials and Methods . (DOC) [file pcbi.1003749.s008.doc]

| **Model** | **WT** | **D816V** | **D816Y** | **D816N** | **D816H** | **V560G** | **V560D** |
| --- | --- | --- | --- | --- | --- | --- | --- |
| **cutoff *r* (Å)** | 2.5 | 2.5 | 2.5 | 2.5 | 3.0 | 2.5 | 3.0 |
| **Number of *reference* structures** | 4-7 /  5-7 | 2-4 / 4-6 | 2-3 / 2-4 | 3-4 / 2-5 | 2-4 / 3-4 | 4-6 / 4-5 | 3-6 / 3-5 |
| **Convergence criterion *c*** | 0.6 / 0.4 | 0.7 / 0.6 | 0.8 / 0.8 | 0.6 / 0.7 | 0.8 / 0.9 | 0.5 / 0.8 | 0.9 / 0.9 |
| **Mean Convergence criterion *c*** | 0.5 | 0.65 | 0.8 | 0.65 | 0.85 | 0.65 | 0. 9 |
