# Supplementary material for: Hotspot Mutations in KIT Receptor Differentially Modulate Its Allosterically Coupled Conformational Dynamics: Impact on Activation and Drug Sensitivity
Source: PLoS Comput Biol. 2014 Jul 31;10(7):e1003749. doi: 10.1371/journal.pcbi.1003749 (PMC4117417; doi:10.1371/journal.pcbi.1003749)
Supplement: Table S4 — The A-loop and the JM-Switch internal H-bond network in KITWT and KITD816V/H/Y/N. Only the H-bonds which time occupancy differs significantly among the simulated models are presented. The residue and its fragment, the side chains – sc – or the backbone – bb, involved in the A-loop and JMR internal H-bonding are shown. (DOC) [file pcbi.1003749.s009.doc]

| JM-Switch | | KIT^WT^ | KIT^D816V^ | KIT^D816H^ | KIT^D816Y^ | KIT^D816N^ |
| --- | --- | --- | --- | --- | --- | --- |
| V559 bb | I571 bb | 98 | 108 | 98 | 110 | 97 |
| E561 bb | V569 bb | 87 | **189** | **120** | **128** | **132** |
| I563 bb | N567 bb | 5 | 5 | 2 | 16 | 5 |

| A-loop | | KIT^WT^ | KIT^D816V^ | KIT^D816H^ | KIT^D816Y^ | KIT^D816N^ |
| --- | --- | --- | --- | --- | --- | --- |
| 816 bb | N819 bb | 21 | 5 | 0 | 6 | 8 |
| 816 bb | D820 bb | 13 | 2 | 0 | 4 | 5 |
| 816 sc | 818 sc/bb | 72 | - | 1 | 0 | 30 |
| 816 sc | 819 sc/bb | 20 | - | 2 | 0 | 14 |
