# Supplementary material for: Hotspot Mutations in KIT Receptor Differentially Modulate Its Allosterically Coupled Conformational Dynamics: Impact on Activation and Drug Sensitivity
Source: PLoS Comput Biol. 2014 Jul 31;10(7):e1003749. doi: 10.1371/journal.pcbi.1003749 (PMC4117417; doi:10.1371/journal.pcbi.1003749)
Supplement: Table S5 — Distance and angle measurements. The distance DJMS between the JM-Switch and the center of mass of residues 847 and 912 in the C-lobe, the angle AJMS drawn by the JM-Switch, the PKD and the C-lobe, and the distance DA between the A-loop and the rest of PKD where measured every 10 ps and averaged upon 2×48-ns or 2×65-ns of productive simulation time. (DOC) [file pcbi.1003749.s010.doc]

|  | **Value** | **WT** | **D816V** | **D816H** | **D816Y** | **D816N** | **V560G** | **V560D** |
| --- | --- | --- | --- | --- | --- | --- | --- | --- |
| **D_JMS_** | mean (Å) | 12.4 / 11.7 | 13.7 / 12.5 | **12.8 / 13.8** | 12.0 / **13.1** | **13.2 /13.2** | **13.6** / 12.1 | **17.7 / 16.6** |
|  | sd(Å) | 0.9 / 1.2 | 1.1 / 1.2 | 1.0 / 1.0 | 0.9 / 1.2 | 1.1 / 1.2 | **1.2 / 1.6** | **2.2 / 1.6** |
|  | min (Å) | 8.6 / 8.4 | **10.7 / 9.3** | **9.8 / 10.0** | **9.6 / 9.4** | **9.5 / 9.2** | **8.4 / 7.9** | **11.8 / 11.3** |
|  | max (Å) | 16.1 / 15.9 | **18.9 / 17.6** | **17.4 / 17.7** | 15.3 / **17.2** | **17.7 / 17.1** | **17.0 / 16.7** | **24.3 / 23.7** |
|  | ‰ **D_JMS_** > 16 Å | 0 / 0 | **44 / 5** | **3 / 23** | 0 / 5 | **14 / 4** | **8 / 1** | 748 / 727 |
|  | ‰ **D_JMS_** < 10 Å | 6 / 68 | 0 / 0 | 0 / 0 | 3 / 3 | 2 / 3 | 33 / 125 | 0 / 0 |
|  | | | | | | |  |  |
| **A_JMS_** | mean (°) | 76.1 / 75.5 | 78.6 / 75.6 | 78.8 / 80.6 | **77.2 / 78.5** | **78.4 / 79.4** | 78.6 / 74.4 | 88.2 / 86.1 |
|  | sd(Å) | 2.1 / 3.5 | 2.7 / 2.4 | 2.1 / 2.3 | 2.1 / 2.8 | 2.0 / 3.5 | 2.2 / 2.3 | 5.0 / 3.1 |
|  | min (°) | 67.7 / 66.3 | 72.2 / 68.5 | 72.4 / 71.2 | **70.8 / 70.3** | **70.6** / 67.7 | 69.3 / 65.3 | 75.5 / 74.8 |
|  | max (°) | 83.7 / 85.1 | 91.6 / 86.3 | 87.3 / 90.3 | 85.0 / **87.0** | **87.4 / 89.5** | 87.1 / 84.2 | 105 / 99 |
|  | ‰ **A_JMS_** > 85° | 0 / 0 | 29 / 1 | 2 / 35 | 0 / **37** | **25 / 299** | 1 / 0 | 691 / 644 |
|  | ‰ **A_JMS_** < 70° | 2 / 36 | 0 / 1 | 0 / 0 | 0 / 0 | 0 / 67 | 0 / 109 | 0 / 0 |
|  | | | | | | |  |  |
| **D_A_** | mean (Å) | 15.2 / 15.3 | 15.3 / **16.3** | 15.3 / 15.5 | 15.2 / 15.0 | 15.1 / 14.9 | **16.3** / 15.3 | 15.3 / 15.4 |
|  | sd(Å) | 0.3 / 0.4 | 0.3 / **0.8** | 0.3 / 0.3 | 0.3 / 0.2 | 0.3 / 0.3 | 0.5 / 0.3 | 0.3 / 0.3 |
|  | min (Å) | 14.4 / 14.3 | 14.4 / 14.9 | 14.7 / 14.8 | 14.4 / 14.2 | 14.1 / 14.1 | 14.8 / 14.4 | 14.4 / 14.5 |
|  | max (Å) | 15.9 / 16.2 | 16.3 / **18.0** | 16.0 / 16.6 | 16.1 / 15.6 | 15.9 / 16.1 | **17.9 / 17.3** | 16.3 / 16.6 |
|  | ‰ D_Aloop_ > 16 Å | 0 / 2 | 2 / **58** | 0 / 8 | 0 / 0 | 0 / 0 | **71** / 1 | 1 / 6 |
|  | ‰ D_Aloop_ < 15 Å | 95 / 95 | 4 / 67 | 11 / 82 | 34 / 45 | 26 / 72 | **0 / 9** | **18 / 10** |
